# A nickel-catalyzed tandem reaction involving cyclic esterification/C–S bond formation for synthesizing 5-oxa-11-thia-benzofluoren-6-ones†

**DOI:** 10.1039/d0ra04367b

**Published:** 2020-07-14

**Authors:** Rongrong Cai, Qicai Wei, Runsheng Xu

**Affiliations:** Department of Biology and Environment, Jiyang College of Zhejiang A&F University Shaoxing 311800 Zhejiang China 20140041@zafu.edu.cn

## Abstract

A nickel-catalyzed tandem reaction involving cyclic esterification/C–S bond formation has been developed. Starting from samples containing 3-(2-hydroxy-phenyl)-acrylic acids with 2-halide-benzenethiols, versatile biologically active 5-oxa-11-thia-benzofluoren-6-one compounds were efficiently synthesized in good to high yields. This new methodology provides an economical approach toward C–S bond formation.

## Abstract 2

A nickel-catalyzed tandem reaction involving cyclic esterification/C–S bond formation has been developed.

## Graphical abstract

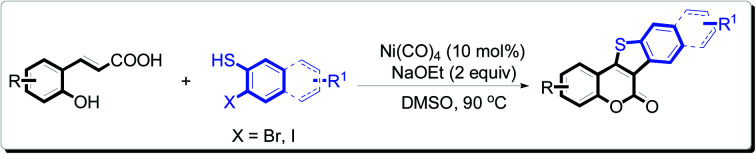

Sulfur-containing organic compounds have been widely applied in syntheses of pharmaceutical and functional materials.^1^ Due to its relatively large atomic radius and high electron density, sulfur displays relatively high reactivity and is easy to modify, at least in theory.^2^ In recent decades, the activation of the C–H bond is considered as one of the most useful C–S bond formation strategies (Scheme 1a). However, compared to C–X (X = I, Br, Cl) cross-coupling, direct C–S bond cross-coupling reactions require harsher conditions and more activated reaction systems (Scheme 1b).^3^ Given the present challenges, the development of more efficient and environmentally friendly chemical processes for drug discovery is required.^4^

**Scheme 1:**
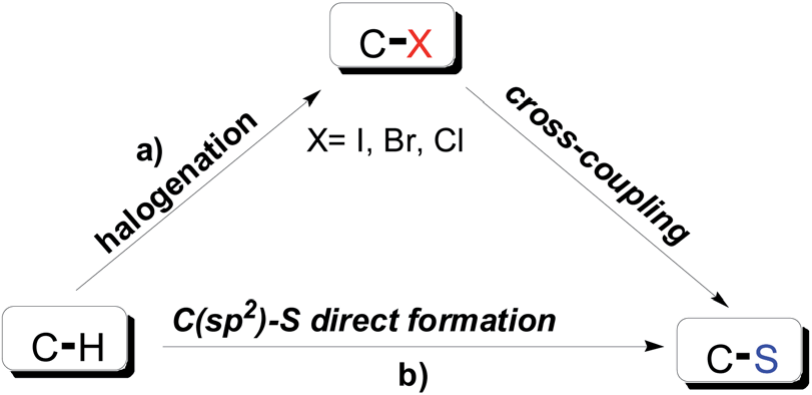
C–S bond formation synthesis approaches.

5-Oxa-11-thia-benzofluoren-6-one constitutes the central core unit of a variety of natural polycyclic lactones with important biological activities, including anticancer, antibacterial, antimyotoxic, and phytoalexin effects.^5^ A wide range of biological properties make 5-oxa-11-thia-benzofluoren-6-ones interesting synthetic targets for chemists. Several synthetic methods have been developed for the construction of this privileged structural unit.^6^ Most of the reported procedures involve multiple steps with moderate overall yields. The starting materials are often not very readily available. And harsh reaction conditions are usually required. In view of these limitations, the development of an efficient strategy for synthesizing 5-oxa-11-thia-benzofluoren-6-ones is highly desirable. Herein, we report a novel nickel-catalyzed tandem reaction involving cyclic esterification/C–S bond formation (Scheme 2). Versatile biologically active 5-oxa-11-thia-benzofluoren-6-one compounds were efficiently synthesized in good to high yields under mild conditions. This new methodology was concluded to provide an economical approach toward C–S bond formation.

**Scheme 2:**
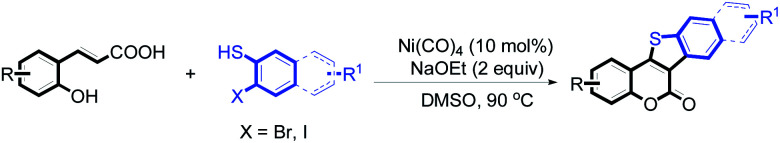
The nickel-catalyzed tandem reaction for cyclic esterification/C–S bond formation.

At first, the reaction conditions were screened based on the model reaction of 3-(2-hydroxy-phenyl)-acrylic acid 1a with 2-iodo-benzenethiol 2a (Table 1). The structure of 3a was confirmed from ^1^H NMR, ^13^C NMR, and HRMS analyses. Various nickel-containing catalysts were tested, and displayed good catalytic activities in the presence of Na_2_CO_3_ (entries 1–7), with the Ni(CO)_4_ catalyst exhibiting the best catalytic efficiency (entry 7). Various bases were also tested, and NaOEt was found to be the optimal base (entry 12), having produced the product 3a with an 83% yield. Better results were also obtained when using a 1 : 1.2 ratio of 1a to 2a than when using a 1 : 1 ratio (entries 10 and 12). Also, under these optimized conditions, the product yield was better when the reaction temperature was 90 °C than when it was 80 °C or 100 °C (entries 12, 13 and 14). Furthermore, the results also showed that the reaction yield was higher when using DMSO as the solvent than when using CHCl_3_ or DMF as the solvent (entries 12, 15 and 16). Thus, the optimum reaction condition was determined to be that involving reacting 1a and 2a in a 1 : 1.2 ratio in the presence of Ni(CO)_4_ (10 mol%) and NaOEt (2 equiv.) in DMSO (5 mL) at 90 °C for 10 hours (Table 1, entry 12).

**Table 1:** Optimization of the reaction conditions^a^

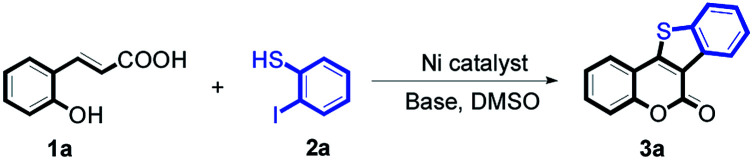				
Entry	Ni catalyst	Base	1a : 2a	3a^b^ (%)
1	NiCl_2_	Na_2_CO_3_	1 : 1	nr
2	NiBr_2_	Na_2_CO_3_	1 : 1	36
3	NiSO_4_	Na_2_CO_3_	1 : 1	44
4	(PCy_3_)_2_NiCl_2_	Na_2_CO_3_	1 : 1	39
5	(DPPE)NiCl_2_	Na_2_CO_3_	1 : 1	55
6	(PPh_3_)_2_NiCl_2_	Na_2_CO_3_	1 : 1	30
7	Ni(CO)_4_	Cs_2_CO_3_	1 : 1	77
8	Ni(CO)_4_	NaOH	1 : 1	65
9	Ni(CO)_4_	Na_2_SO_4_	1 : 1	50
10	Ni(CO)_4_	NaOEt	1 : 1	54
11	Ni(CO)_4_	NEt_3_	1 : 1	46
12	Ni(CO)_4_	NaOEt	1 : 1.2	83
13	Ni(CO)_4_	NaOEt	1 : 1.2	68^c^
14	Ni(CO)_4_	NaOEt	1 : 1.2	75^d^
15	Ni(CO)_4_	NaOEt	1 : 1.2	73^e^
16	Ni(CO)_4_	NaOEt	1 : 1.2	54^f^

a Unless otherwise noted, reaction conditions were 1a (0.5 mmol), 2a (0.5 mmol), nickel catalyst (10 mol%), base (2 equiv.), DMSO (5 mL), 90 °C, and a reaction time of 10 h.

b Isolated yield.

c At 80 °C.

d At 100 °C.

e In CHCl_3_.

f In DMF.

Next, a wide array of 3-(2-hydroxy-phenyl)-acrylic acids 1 and 2-iodo-benzenethiols 2 were subjected to this reaction, and provided the products 3 with good to excellent yields (69–89%, Table 2). 3-(2-Hydroxy-phenyl)-acrylic acids 1 bearing each an electron-donating group (Me and MeO) demonstrated better activity levels than did those bearing each an electron-withdrawing group (F, Cl, and Br). 2-Iodo-benzenethiols 2 bearing each an electron-withdrawing group also demonstrated better activity than did those bearing each an electron-donating group. Notably, use of very strong electron-withdrawing groups, such as trifluoromethyl and nitro groups, failed to lead to the corresponding products.

**Table 2:** Nickel-catalyzed tandem reactions of 3-(2-hydroxy-phenyl)-acrylic acids 1 and 2-iodo-benzenethiols 2, each involving cyclic esterification/C–S bond formation^a^

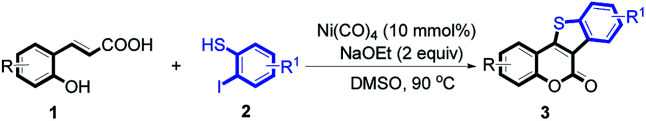				
Entry	R	R^1^	3	Yield^b^
1	H	H	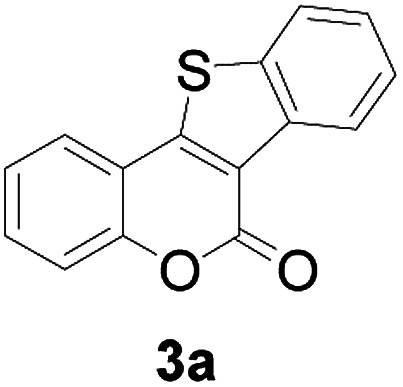	83
2	5-CH_3_	H	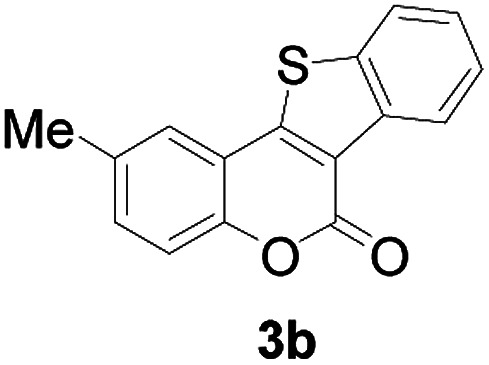	84
3	5-CH_3_	Naphthyl	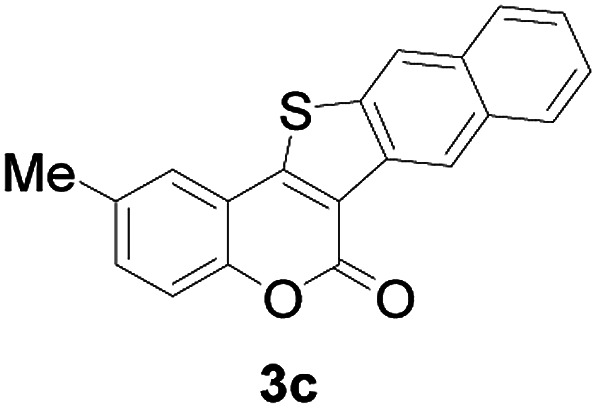	86
4	4-CH_3_O	H	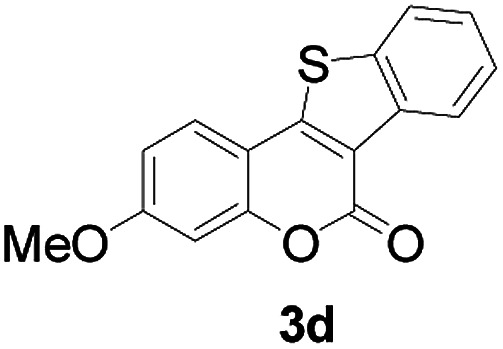	89
5	4-CH_3_O	4,5-diCH_3_O	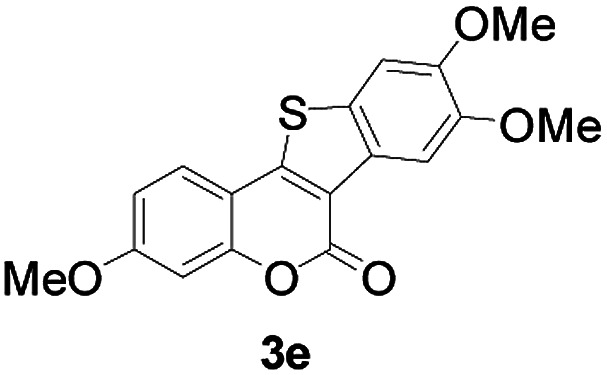	76
6	4-CH_3_O	Naphthyl	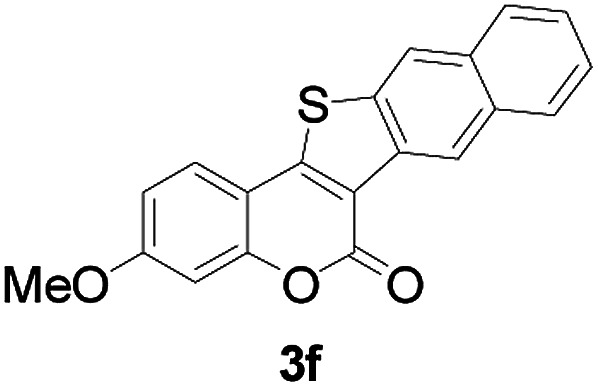	81
7	5-F	H	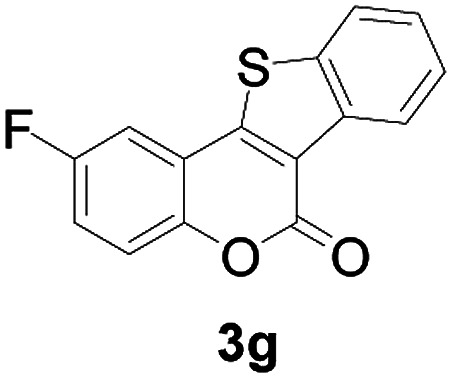	71
8	5-Cl	H	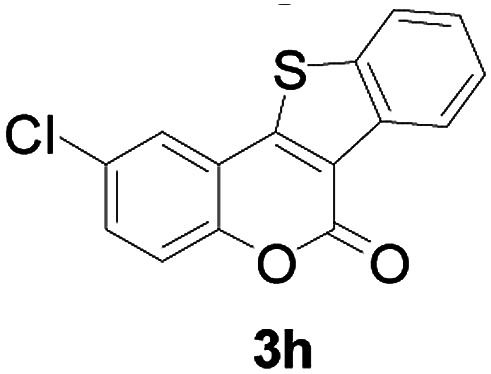	75
9	5-Br	H	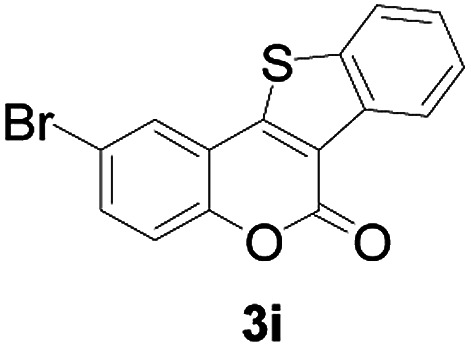	69
10	4,5-diCH_3_	H	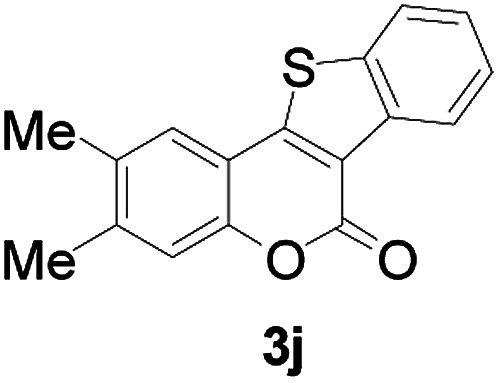	74
11	4,5-diCH_3_O	Naphthyl	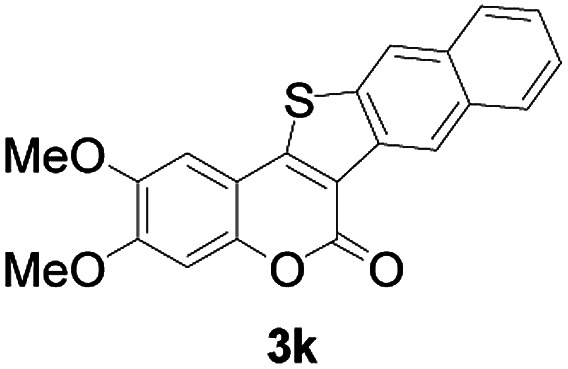	72

a Unless otherwise noted, reaction conditions were 1 (0.5 mmol), 2 (0.6 mmol), Ni(CO)_4_ (10 mol%), NaOEt (2 equiv.), DMSO (5 mL), 90 °C and a reaction time of 10 h.

b Isolated yield.

Furthermore, other 3-(2-hydroxy-phenyl)-acrylic acids 1 with 2-bromo-benzenethiols 4 also successfully provided the corresponding products (Table 3). 3-(2-Hydroxy-4,5-dimethoxy-phenyl)-acrylic acid displayed a moderate reactivity with chlorobenzene, and the corresponding yield was 64% (entry 4). Furthermore, to our delight, reactants with more substituents also proceeded smoothly (entry 6).

**Table 3:** Nickel-catalyzed tandem reactions of 3-(2-hydroxy-phenyl)-acrylic acids 1 with 2-bromo-benzenethiols 4, each involving cyclic esterification/C–S bond formation^a^

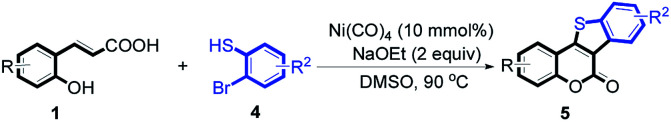				
Entry	R	R^2^	3	Yield^b^
1	H	H	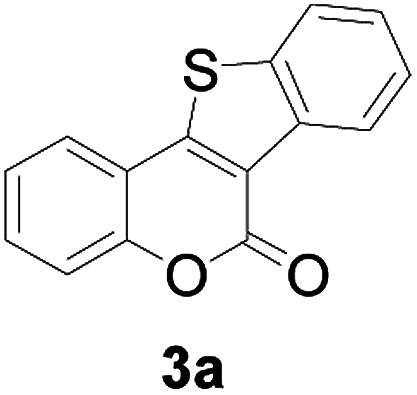	73
2	4-CH_3_O	H	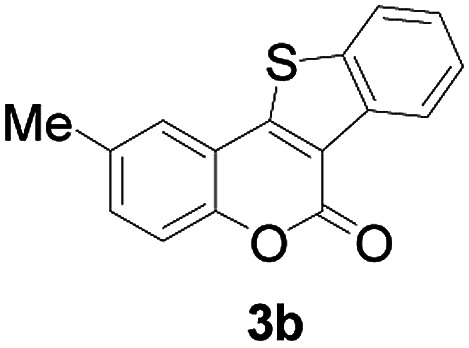	69
3	5-Cl	H	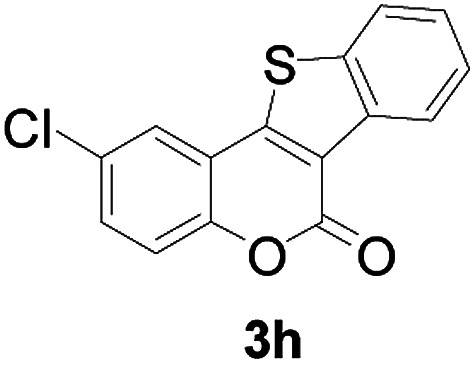	75
4	4,5-diCH_3_	H	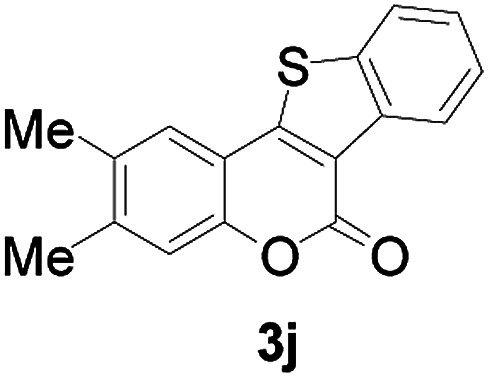	64
5	4,5-diCH_3_O	H	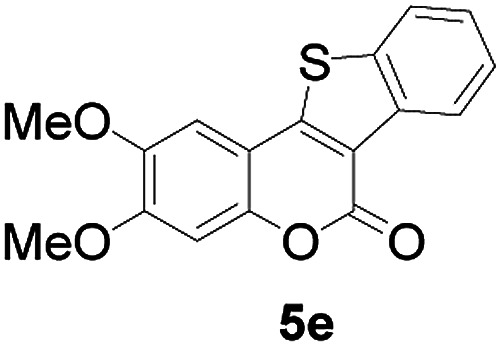	60
6	4,5-diCH_3_O	Naphthyl	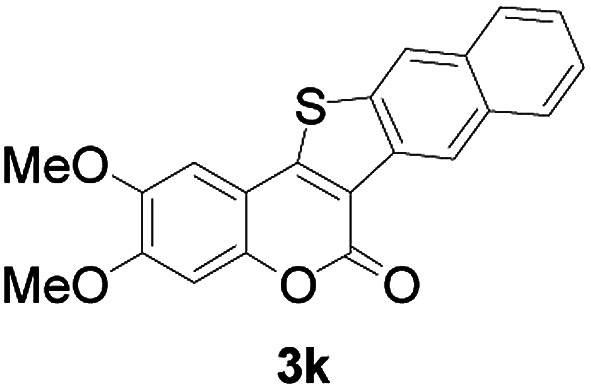	52

a Unless otherwise noted, reaction conditions were 1 (0.5 mmol), 3 (0.6 mmol), Ni(CO)_4_ (10 mol%), NaOEt (2 equiv.), DMSO (5 mL), 90 °C, and a reaction time of 10 h.

b Isolated yield.

## Conclusions

In summary, we have reported a nickel–catalyzed tandem reaction involving cyclic esterification/C–S bond formation. Starting from samples of 3-(2-hydroxy-phenyl)-acrylic acids with 2-halide-benzenethiols, versatile biologically active 5-oxa-11-thia-benzofluoren-6-one compounds were efficiently synthesized in good to high yields. This new methodology provides an economical approach toward C–S bond formation.

## Conflicts of interest

There are no conflicts to declare.

## Supplementary Material

RA-010-D0RA04367B-s001
