# Supplementary material for: A nickel-catalyzed tandem reaction involving cyclic esterification/C–S bond formation for synthesizing 5-oxa-11-thia-benzofluoren-6-ones
Source: RSC Adv. 2020 Jul 14;10(44):26414–7. doi: 10.1039/d0ra04367b (PMC9055437; doi:10.1039/d0ra04367b)
Supplement: RA-010-D0RA04367B-s001 [file RA-010-D0RA04367B-s001.pdf]

Electronic Supplementary Material (ESI) for RSC Advances.
This journal is © The Royal Society of Chemistry 2020

Electronic Supplementary Information

Nickel–Catalyzed Tandem Reaction of Cyclic Esterification/C–S Bond Formation For Synthesis of 5-Oxa-11-thia-benzofluoren-6-ones

Rongrong Cai, Qicai Wei and Runsheng Xu,*

Department of Biology and Environment, Jiyang College of Zhejiang A＆F University, Shaoxing, Zhejiang 311800, China

* Correspondence: 20140041@zafu.edu.cn; Tel.: +86-0575-87765010

Contents

Experimental Details... S2

Analytical Datas...S2

Spectrums……………………………………………………………………………………………….......................... S8

Experimental Details

Materials

All reagents used in the experiment were obtained from commercial sources and used without further purification. Solvents for chromatography were of technical grade and distilled prior to use. Solvent mixtures were understood as volume/volume. Chemical yields refer to pure isolated substances. Catalysts were purchased from Alfa Aesar (analytical reagent). Thin layer chromatography (TLC) employed glass 0.25 mm silica gel plates with an F-254 indicator, visualized by irradiation with UV light. The NMR spectra were recorded on a Bruker AVANCE III-400 spectrometer at 400 MHz and 100 MHz for ^{1}H and ^{13}C NMR in $CDCl_3$, respectively. The NMR chemical shift was reported in ppm relative to 7.26 and 77 ppm of $CDCl_3$ as the standards of ^{1}H and ^{13}C NMR, respectively. The mass spectra were performed on a Bruker Esquire 3000 plus mass spectrometer equipped with an ESI interface and ion trap analyzer. The ESI-HRMS was tested on a Bruker 7-tesla FT-ICR MS equipped with an electrospray source.

General Synthesis Methods of 3a–5f

A solution of 3-(2-hydroxy-phenyl)-acrylic acids **1** (0.5 mmol), 2-halide-benzenethiols **2** or **4** (0.6 mmol), $Ni(CO)_4$ (10 mol%, 8.5 mg), and NaOEt (2 equiv, 68 mg) in DMSO (5 mL) was stirred under air. After stirred at 90 °C for 10 h, it was cooled to room temperature. Then the reaction mixture was quenched with saturated salt water (10 mL). After that, the solution was extracted with ethyl acetate (3 × 10 mL), and then washed with saturated Na_2CO_3 solution. The organic layers were combined and dried by Na_2SO_4 and concentrated in vacuo. The pure product **3** or **5** (69-89% yield) was afforded by flash column chromatography on silica gel (cyclohexane/ethyl acetate).

Analytical Datas

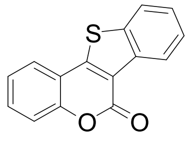

3a

Benzo[4,5]furo[3,2-c]chromen-6-one (3a) white solid, mp 181-182 °C, was afforded by flash column chromatography on silica gel (cyclohexane/ethyl acetate = 5:1);

^{1}H NMR (500 MHz, $CDCl_3$): δ 8.15 (dd, J_1 = 2.4 Hz, J_2 = 6.6 Hz, 1H), 8.05 (dd, J_1 = 1.5 Hz, J_2 = 7.8 Hz,

1H), 7.68 (dd, J_1 = 1.7 Hz, J_2 = 6.9 Hz, 1H), 7.60-7.64 (m, 1H), 7.41-7.52 (m, 4H);

^{13}C NMR (125 MHz, $CDCl_3$): δ 160.0, 158.1, 155.6, 153.7, 131.9, 126.8, 125.2, 124.7, 123.5, 121.9, 117.5, 112.7, 111.8, 105.9 (one peak is missing due to overlap);

IR ($CHCl_3$): 2918, 2849, 1736, 1628, 1498, 1452, 1371, 1320, 1192, 1096, 1083, 1032, 972, 912, 889, 780, 744 cm^{-1};

HRMS (+ESI) Calcd for $C_{15}H_9O_2S$ [M+H]$^+$: 253.0320, found 253.0325.

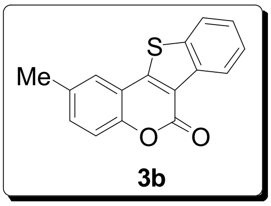

2-Methyl-5-oxa-11-thia-benzo[a]fluoren-6-one (3b) yellow solid, mp 151-153 °C, was afforded by flash column chromatography on silica gel (cyclohexane/ethyl acetate = 5:1);

^{1}H NMR (500 MHz, $CDCl_3$): δ 8.07-8.18 (m, 1H), 7.80 (br s, 1H), 7.59-7.70 (m, 1H), 7.32-7.51 (m, 4H), 2.48 (s, 3H);

^{13}C NMR (125 MHz, $CDCl_3$): δ 160.0, 158.2, 155.5, 151.9, 134.5, 133.0, 126.6, 125.1, 123.5, 121.8, 121.5, 117.2, 112.3, 111.7, 105.8, 20.9;

IR ($CHCl_3$): 2920, 2850, 1713, 1635, 1570, 1447, 1358, 1320, 1161, 1097, 1077, 1009, 982, 817, 776, 748, 737,669, 656 cm^{-1};

HRMS (+ESI) Calcd for $C_{16}H_{11}O_2S$ [M+H]$^+$: 266.0403, found 266.0404.

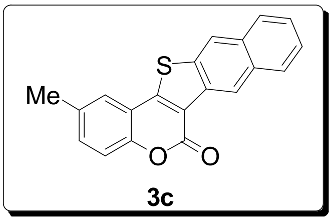

2-Methyl-5-oxa-13-thia-dibenzo[a,h]fluoren-6-one (3c) yellow solid, mp 151-153 °C, was afforded by flash column chromatography on silica gel (cyclohexane/ethyl acetate = 5:1);

^{1}H NMR (500 MHz, $CDCl_3$): δ 8.59 s, 1H), 7.94-8.06 (m, 3H), 7.86 (s, 1H), 7.42-7.56 (m, 4H), 2.51 (s, H);

^{13}C NMR (125 MHz, $CDCl_3$): δ 162.4, 158.3, 153.9, 152.4, 35.7, 134.7, 133.7, 132.1, 128.5, 127.9, 126.2, 125.3, 123.4, 121.8, 20.4, 117.3, 112.1, 107.7, 105.2, 20.9 ;

IR ($CHCl_3$): 2923, 2853, 1729, 714, 1613, 1594, 1506, 1464, 1456, 1367, 1261, 1210, 805, 771, 666,

45 cm^{-1};

HRMS (+ESI) Calcd for $C_{20}H_{13}O_2S$ [M+H]$^+$: 316.6560, Found 316.6562.

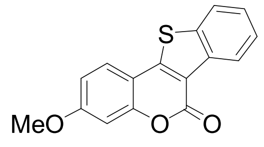

3d

3-Methoxy-5-oxa-11-thia-benzo[a]fluoren-6-one (3d) yellow solid, mp 188-189 °C, was afforded by flash column chromatography on silica gel (cyclohexane/ethyl acetate = 5:1);

^{1}H NMR (500 MHz, $CDCl_3$): δ 8.04–8.14 (m, 1H), 7.91 (dd, J_1= 1.9 Hz, J_2 = 7.3 Hz, 1H), 7.57-7.67 (m, H), 7.39-7.47 (m, 2H), 6.94-7.02 (m, 2H), 3.91 (s, 3H);

^{13}C NMR 125 MHz, $CDCl_3$): δ 163.0, 160.6, 158.3, 155.5, 155.2, 126.0, 125.0, 23.5, 122.8, 121.4, 113.0, 111.4, 105.8, 103.3, 101.3, 55.7;

IR ($CHCl_3$): 2921, 2851, 1729, 1613, 1600, 1447, 1427, 1367, 1275, 254, 1095, 1023, 986, 945, 855, 774, 753, 746 cm^{-1};

HRMS (+ESI) calcd for $C_{16}H_{11}O_3S$ [M+H]$^+$: 282.0353, found 282.0355.

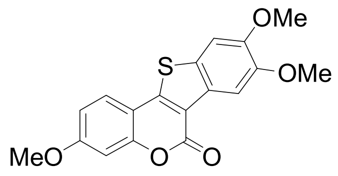

3e

3,8,9-Trimethoxy-5-oxa-11-thia-benzo[a]fluoren-6-one (3e) yellow solid, mp 230-233 °C , was afforded by flash column chromatography on silica gel (cyclohexane/ethyl acetate = 5:1);

^{1}H NMR (500 MHz, $CDCl_3$): δ 7.85 (d, J = 8.4 Hz, 1H), 7.51 (s, 1H), 7.13 (s, 1H), 6.94–7.04 (m 2H), 4.00 (s, 3H), 3.96 (s, 3H), 3.90 (s, 3H);

^{13}C NMR (125 MHz, $CDCl_3$): δ 162.4, 159.7, 158.8, 154.9, 149.9, 149.0, 147.9, 122.3, 115.5, 113.0, 106.3, 103.8, 102.2, 101.4, 95.5, 56.5, 56.4, 55.8;

IR ($CHCl_3$): 2924, 2853, 1741, 1628, 1608, 1494, 1464, 1411, 1278, 1214, 1024, 837, 804, 765 cm^{-1};

HRMS (+ESI) Calcd for $C_{18}H_{15}O_5S$ [M+H]$^+$: 342.0564, found 342.0566.

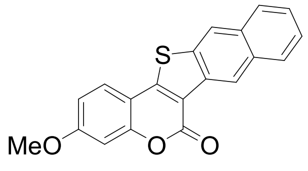

3f

3-Methoxy-5-oxa-13-thia-dibenzo[a,h]fluoren-6-one (3f) yellow solid, mp: 197-200 °C m, was afforded by flash column chromatography on silica gel (cyclohexane/ethyl acetate = 5:1);

^{1}H NMR (500 MHz, $CDCl_3$): δ 8.55 (s, 1H), 7.87-8.12 (m, 4H), 7.43-7.60 (m, 2H), 6.92-7.10 (m, 2H), 3.93 (s, 3H);

^{13}C NMR (125 MHz, $CDCl_3$): δ 163.5, 162.9, 158.2, 156.1, 153.8, 131.8, 131.4, 128.3, 127.9, 125.9, 125.2, 123.4, 123.2, 119.8, 113.2, 107.5, 105.6, 102.6, 101.4, 55.8 ;

IR ($CHCl_3$): 3419, 2922, 2851, 1743, 1613, 1465, 1206, 1161, 1025, 937, 768, 746, 702 cm^{-1};

HRMS (+ESI) Calcd for $C_{20}H_{13}O_3S$ $[M+H]^+$: 332.0507, found 332.0512.

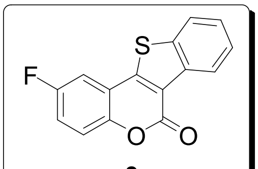

3g

2-Fluoro-5-oxa-11-thia-benzo[a]fluoren-6-one (3g) yellow solid, mp 198-201 °C, was afforded by flash column chromatography on silica gel (cyclohexane/ethyl acetate = 5:1);

^{1}H NMR (500 MHz, $CDCl_3$): δ 8.15 (d, *J* = 7.4 Hz, 1H), 7.62-7.74 (m, 2H), 7.45-7.55 (m, 3H), 7.29-7.34 (m, 1H);

^{13}C NMR (125 MHz, $CDCl_3$): δ 159.0 (d, *J* = 2.8 Hz), 158.9 (d, *J* = 245.7 Hz), 157.6, 155.7, 149.8 (d, *J* = 1.6 Hz), 127.2, 126.4, 123.2, 122.0, 119.4 (d, *J* = 34.7 Hz), 119.3, 113.4 (d, *J* = 9.8 Hz), 111.8, 107.4 (d, *J* = 25.8 Hz), 106.6;

IR ($CHCl_3$): 2920, 2850, 1763, 1734, 1567, 1451, 1401, 1257, 1157, 1094, 1066, 996, 861, 821, 774, 748, 667 cm^{-1};

HRMS (+ESI) Calcd for $C_{15}H_8FO_2S$ $[M+H]^+$: 270.0153, found 270.0151.

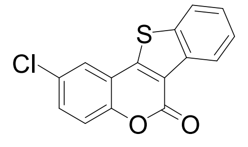

3h

2-Chloro-5-oxa-11-thia-benzo[a]fluoren-6-one (3h) yellow solid, mp 219-220 °C, was afforded by

flash column chromatography on silica gel (cyclohexane/ethyl acetate = 5:1);

^{1}H NMR (500 MHz, $CDCl_3$): δ 8.14 (dd, J_1 = 1.2 Hz, J_2 = 7.9 Hz, 1H), 8.01 (d, J = 2.5 Hz, 1H), 7.68 (d, J = 7.6 Hz, 1H), 7.55 (dd, J_1 = 2.5 Hz, J_2 = 8.9 Hz, 1H), 7.47-7.52 (m, 2H), 7.45 (d, J = 8.9 Hz, 1H);

^{13}C NMR (125 MHz, $CDCl_3$): δ 158.5, 157.4, 155.6, 151.8, 131.8, 130.2, 127.1, 125.4, 123.1, 121.9, 121.2, 118.8, 113.6, 111.8, 106.5;

IR ($CHCl_3$): 2919, 2850, 1760, 1732, 1556, 1447, 1416, 1162, 1094, 1068, 981, 870, 822, 774, 750, 662 cm^{-1};

HRMS (+ESI) Calcd for $C_{15}H_8ClO_2S$ [M+H]$^+$: 285.9857, found 285.9862

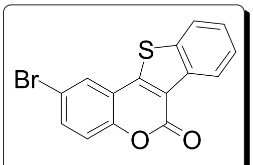

3i

2-Bromo-5-oxa-11-thia-benzo[a]fluoren-6-one (3i) yellow solid, mp 215-217 °C, was afforded by flash column chromatography on silica gel (cyclohexane/ethyl acetate = 5:1);

^{1}H NMR (500 MHz, $CDCl_3$): δ 8.18 (d, J = 2.3 Hz, 1H), 8.13-8.16 (m, 1H), 7.67-7.71 (m, 2H), 7.46-7.55 (m, 2H), 7.40 (d, J = 8.8 Hz, 1H);

^{13}C NMR (125 MHz, $CDCl_3$): δ 158.4, 157.3, 155.6, 152.3, 134.6, 127.2, 125.4, 124.3, 123.1, 121.9, 119.1, 117.4, 114.1, 111.8, 106.5;

IR ($CHCl_3$): 2921, 2851, 1757, 1734, 1447, 1162, 979, 872, 820, 775, 750, 665 cm^{-1};

HRMS (+ESI) Calcd for $C_{15}H_8BrO2S$ [M+H]$^+$: 329.9352, found 329.9348.

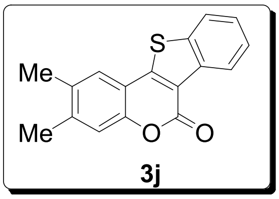

3j

2,3-Dimethyl-5-oxa-11-thia-benzo[a]fluoren-6-one (3j) yellow solid, mp 208-210 °C, was afforded by flash column chromatography on silica gel (cyclohexane/ethyl acetate = 5:1);

^{1}H NMR (500 MHz, $CDCl_3$): δ 8.03-8.14 (m, 1H), 7.67 (s, 1H), 7.56-7.64 (m, 1H), 7.36-7.50 (m, 2H), 7.21 (s, 1H), 2.34 (s, 6H);

^{13}C NMR (125 MHz, $CDCl_3$): δ 160.2, 158.3, 155.2, 152.1, 142.1, 133.5, 126.2, 124.9, 123.6, 121.6, 121.5, 117.8, 111.5, 109.9, 104.8, 20.4, 19.2;

IR ($CHCl_3$): 2917, 2849, 1741, 1638, 1448, 1375, 1177, 1095, 1055, 866, 776, 749, 737 cm^{-1};

HRMS (+ESI) Calcd for $C_{17}H_{13}O_2S$ $[M+H]^+$: 280.0560, found 286.0558.

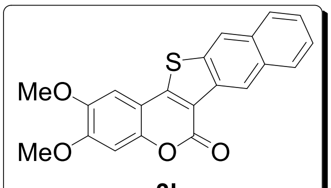
3k

2,3-Dimethoxy-5-oxa-13-thia-dibenzo[a,h]fluoren-6-one (3k) yellow solid, was afforded by flash column chromatography on silica gel (cyclohexane/ethyl acetate = 5:1);

^1H NMR (500 MHz, $CDCl_3$): δ 8.54 (s, 1H), 7.94-8.05 (m, 3H), 7.50-7.55 (m, 2H), 7.39 (s, 1H), 7.00 (s, 1H), 4.04 (s, 3H), 3.99 (s, 3H);

^{13}C NMR (125 MHz, $CDCl_3$): δ 162.9, 158.5, 153.8, 153.6, 150.4, 146.9, 131.8, 131.5, 128.4, 127.9, 126.0, 125.3, 123.6, 119.9, 114.1, 107.5, 104.4, 102.0, 100.6, 56.6, 56.6;

IR ($CHCl_3$): 2960, 2925, 2854, 138, 1732, 1519, 1456, 1276, 1261, 1095, 1020, 800, 675, 664 cm^{-1};

HRMS (+ESI) Calcd for $C_{21}H_{15}O_4S$ $[M+H]^+$: 362.0615, found 362.0617.

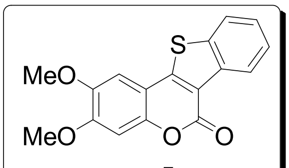
5e

2,3-Dimethoxy-5-oxa-11-thia-benzo[a]fluoren-6-one (5e) yellow solid, mp 227-230 °C, was afforded by flash column chromatography on silica gel (cyclohexane/ethyl acetate = 5:1);

^1H NMR (500 MHz, $CDCl_3$): δ 8.09 (dd, J_1= 3.4 Hz, J_2 = 5.7 Hz, 1H), 7.62 (dd, J_1= 3.0 Hz, J_2 = 6.2 Hz, 1H), 7.44 (d, J = 3.3 Hz, 1H), 7.43 (d, J = 3.1 Hz, 1H), 7.35 (s, 1H), 6.99 (s, 1H), 4.02 (s, 3H), 3.97 (s, 3H);

^{13}C NMR (125 MHz, $CDCl_3$): δ 160.5, 158.4, 155.1, 152.9, 149.6, 146.7, 126.1, 125.0, 123.6, 121.5, 111.3, 104.5, 103.6, 101.7, 100.5, 56.3, 56.3;

IR ($CHCl_3$): 2917, 2849, 1722, 1515, 1459, 1444, 1270 cm^{-1};

HRMS (+ESI) Calcd for $C_{17}H_{13}O_4S$ $[M+H]^+$: 299.0380, found 2990382.

Spectrums

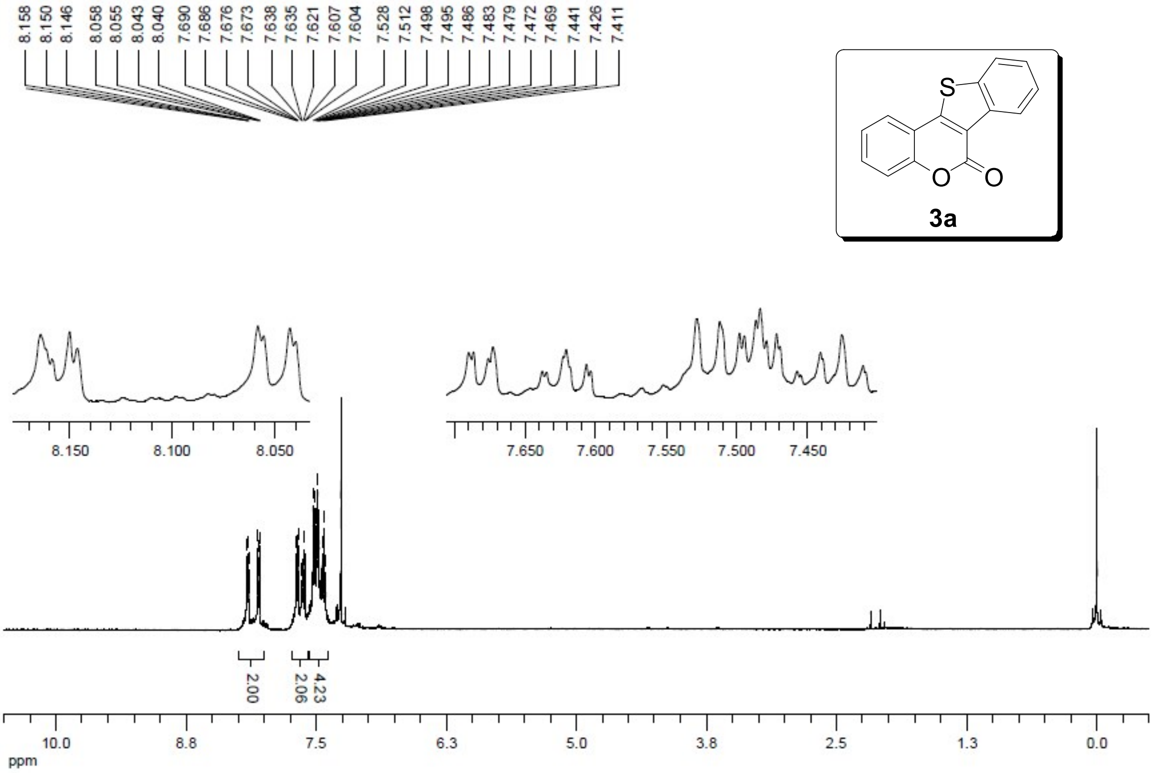

Figure 1. **3a** ^{1}H NMR

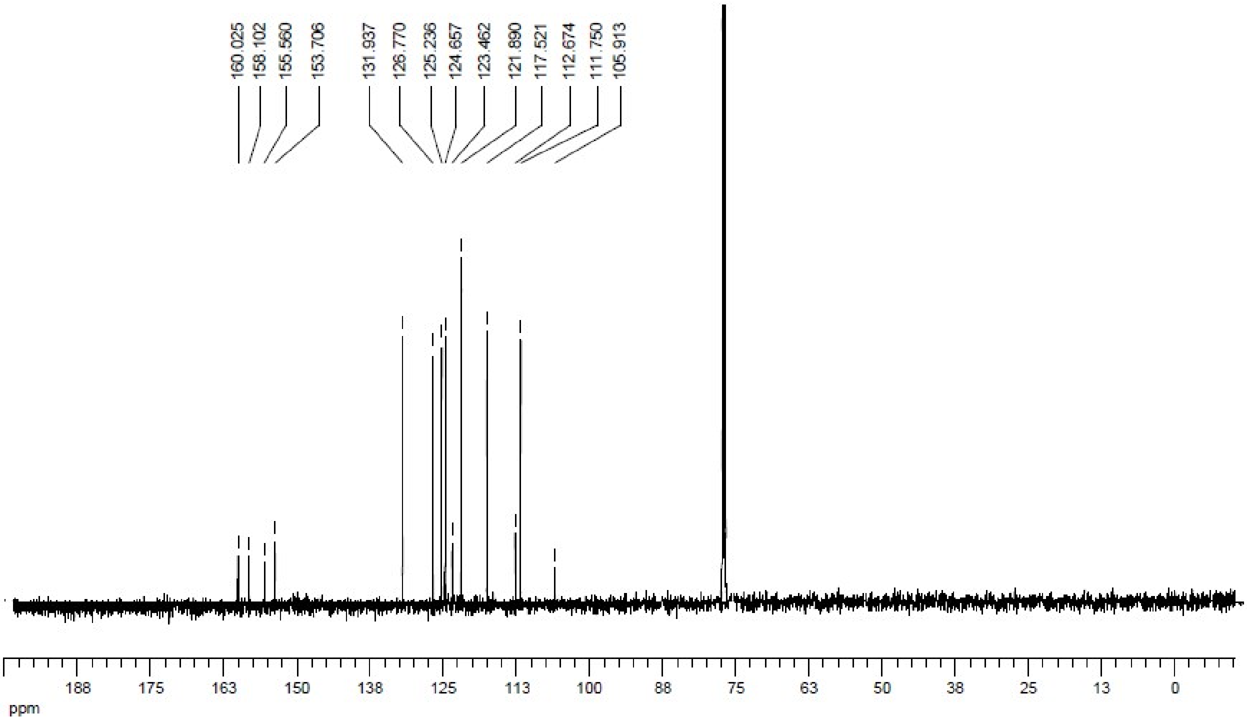

Figure 2. **3a** ^{13}C NMR

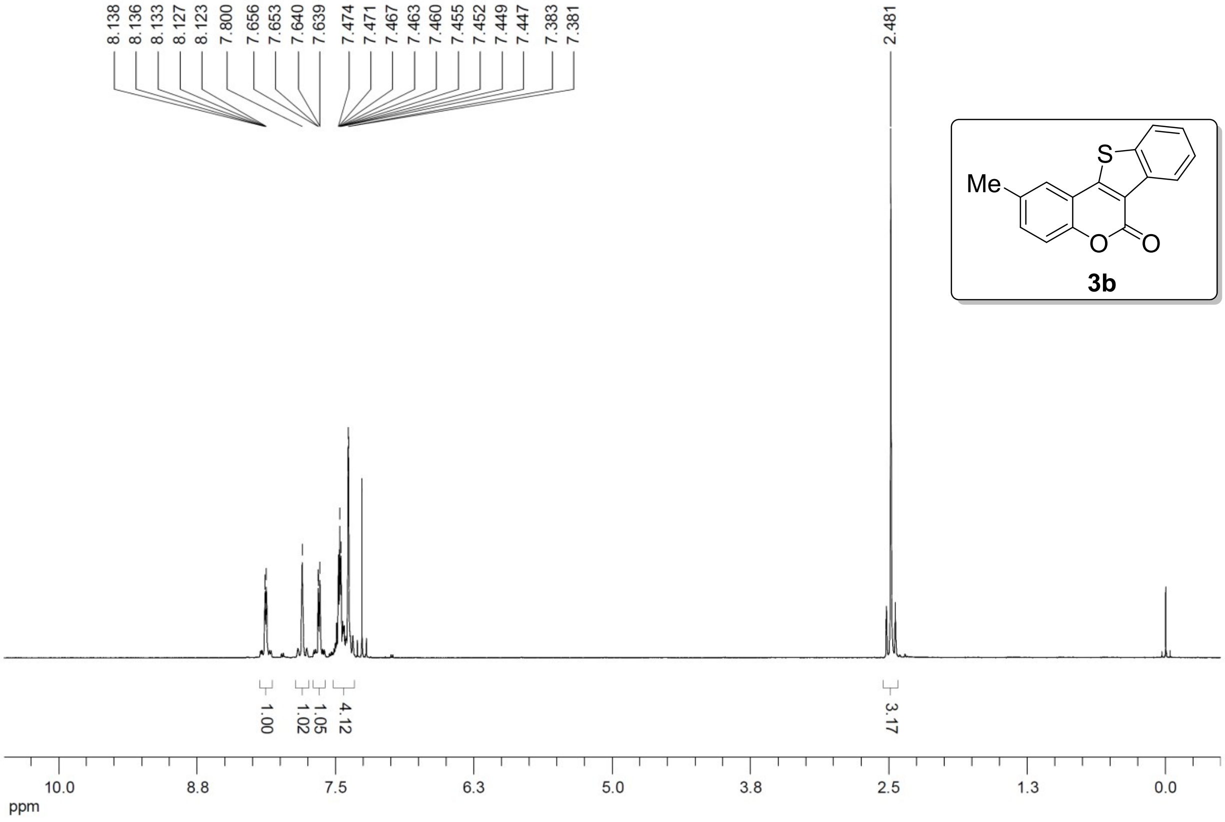

Figure 3. **3b** ^{1}H NMR

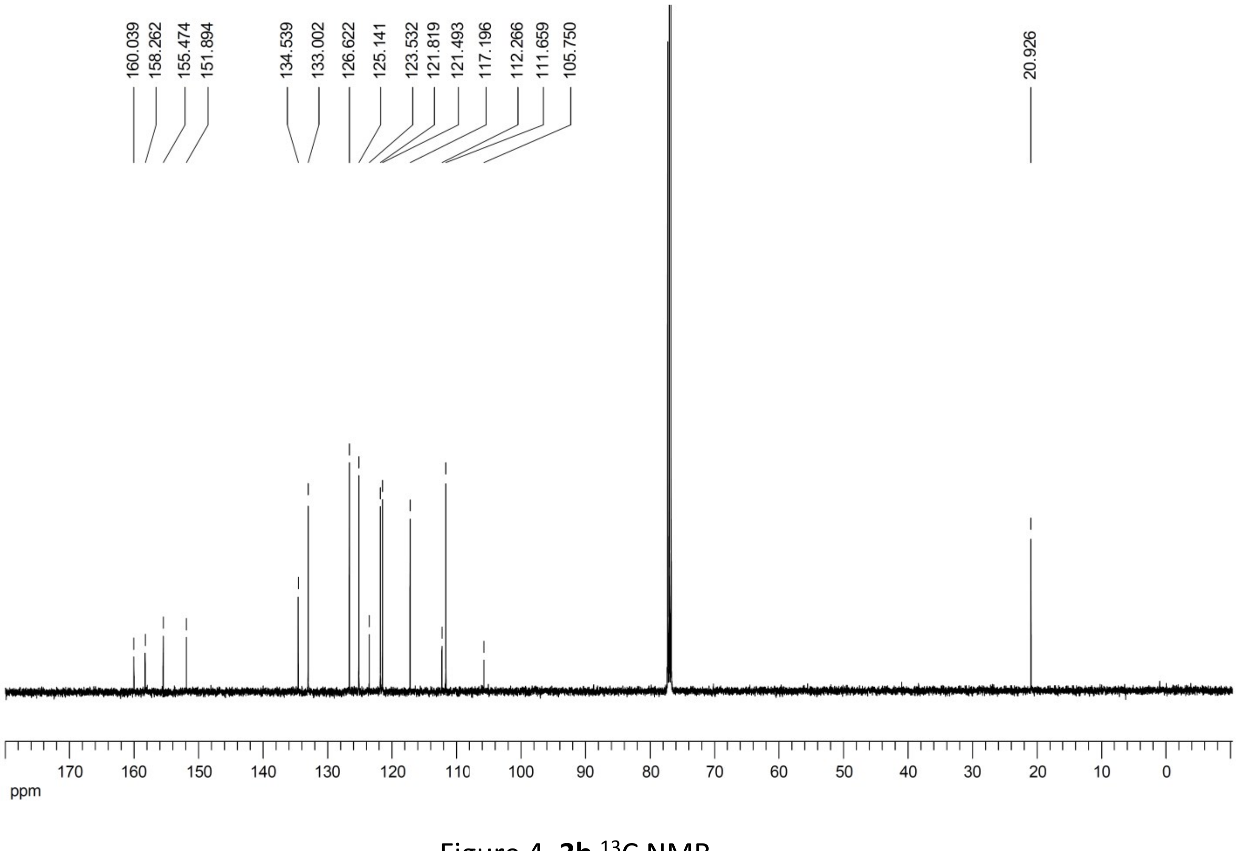

Figure 4. **3b** ^{13}C NMR

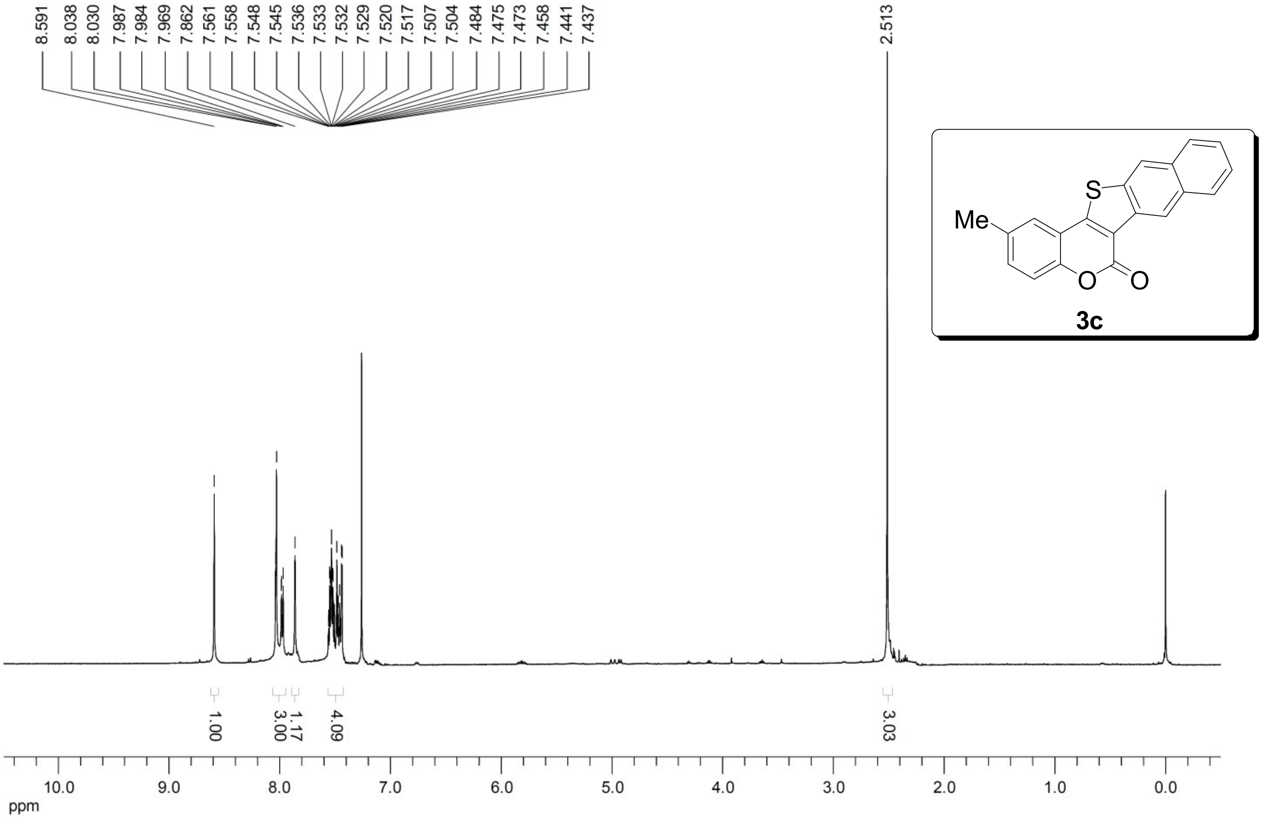

Figure 5. **3c** ^{1}H NMR

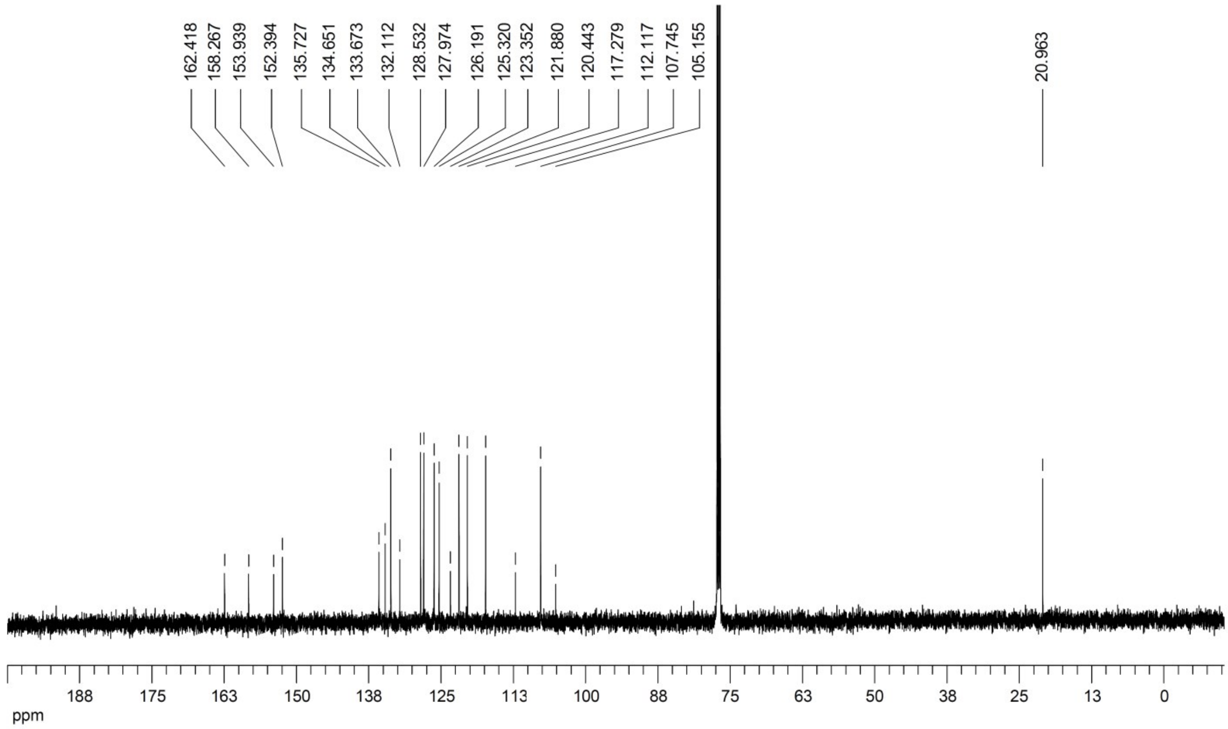

Figure 6. **3c** ^{13}C NMR

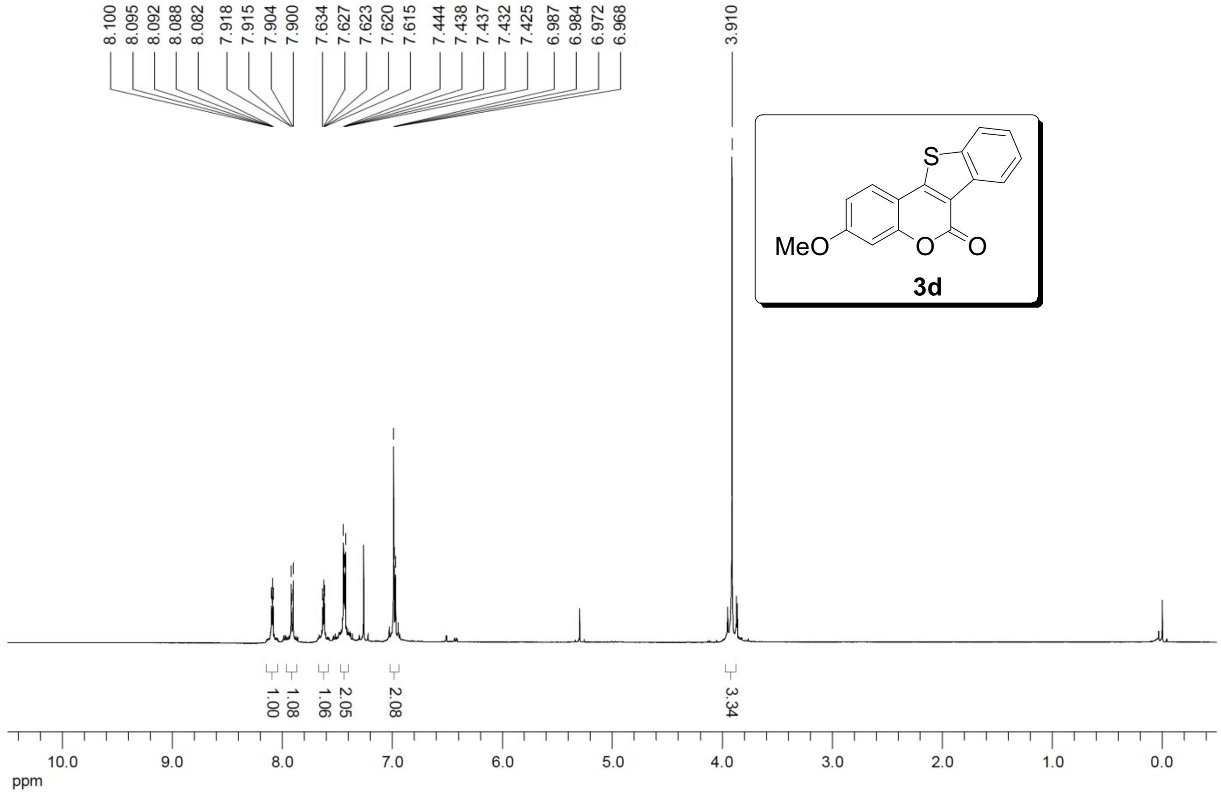

Figure 7. **3d** ^{1}H NMR

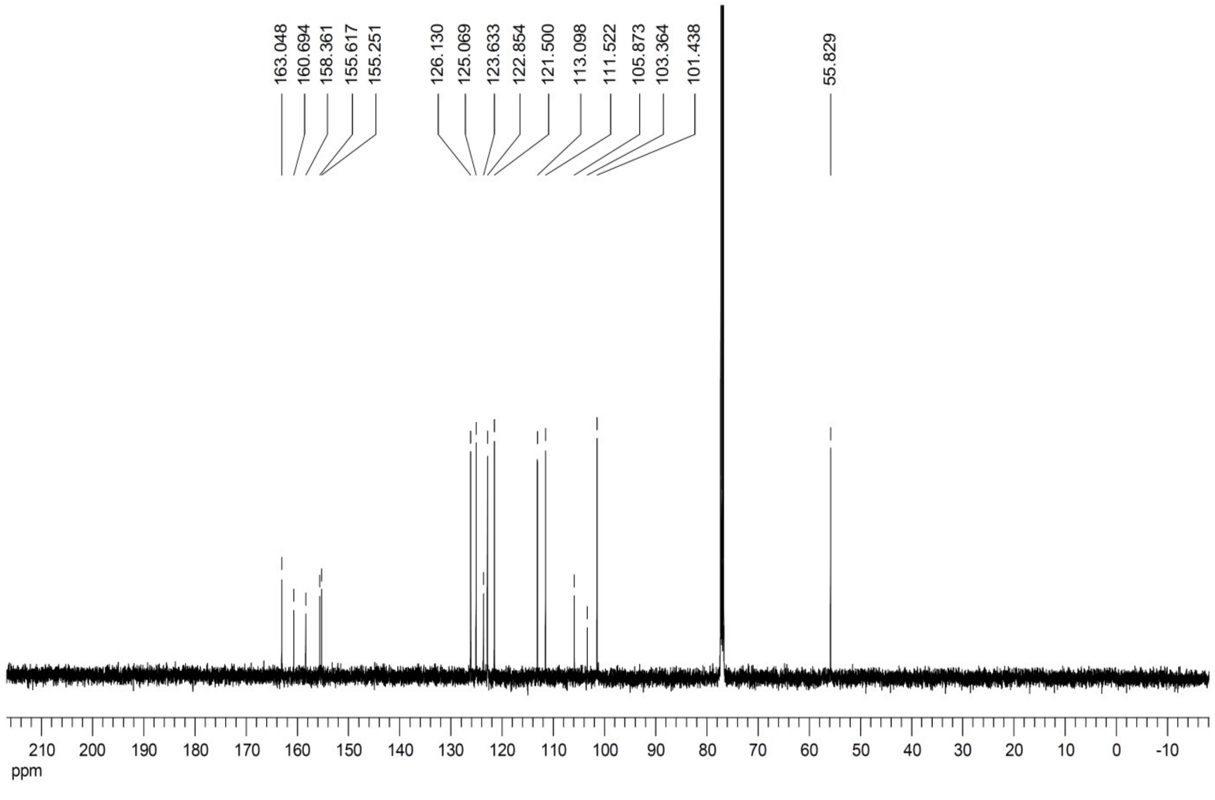

Figure 8. **3d** ^{13}C NMR

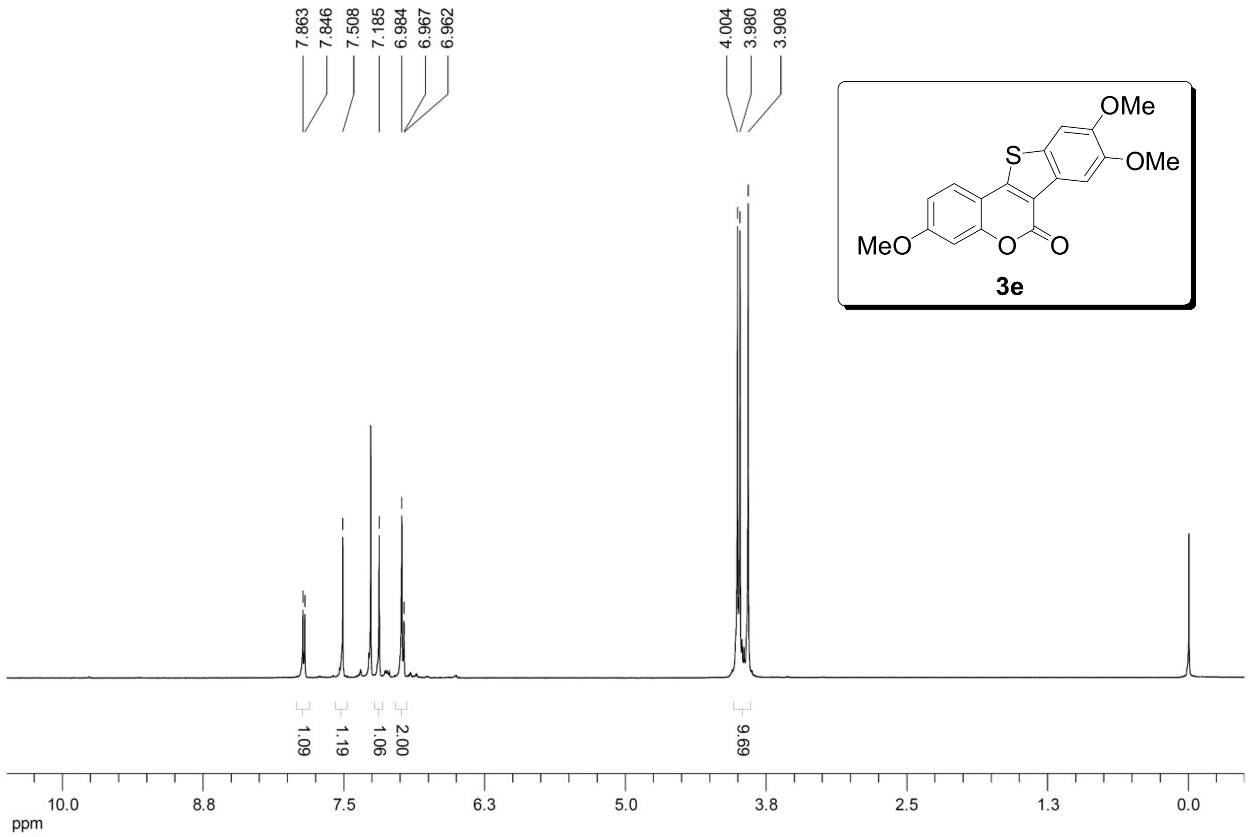

Figure 9. **3e** ^{1}H NMR

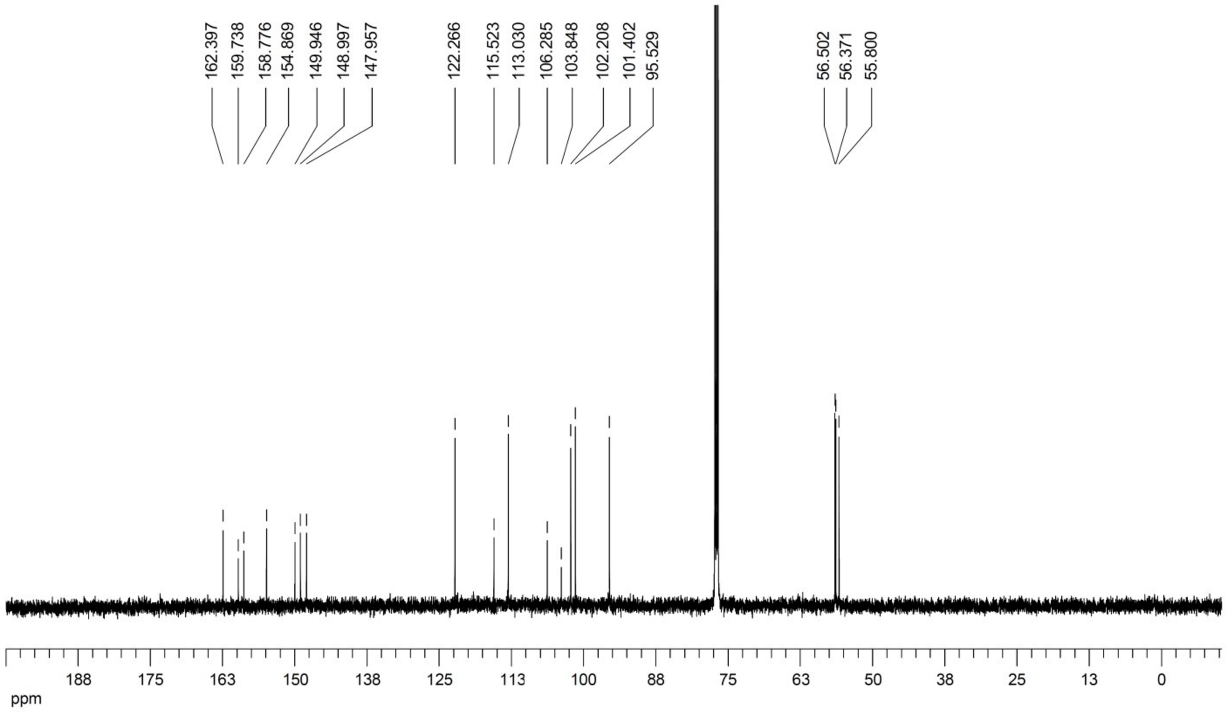

Figure 10. **3e** ^{13}C NMR

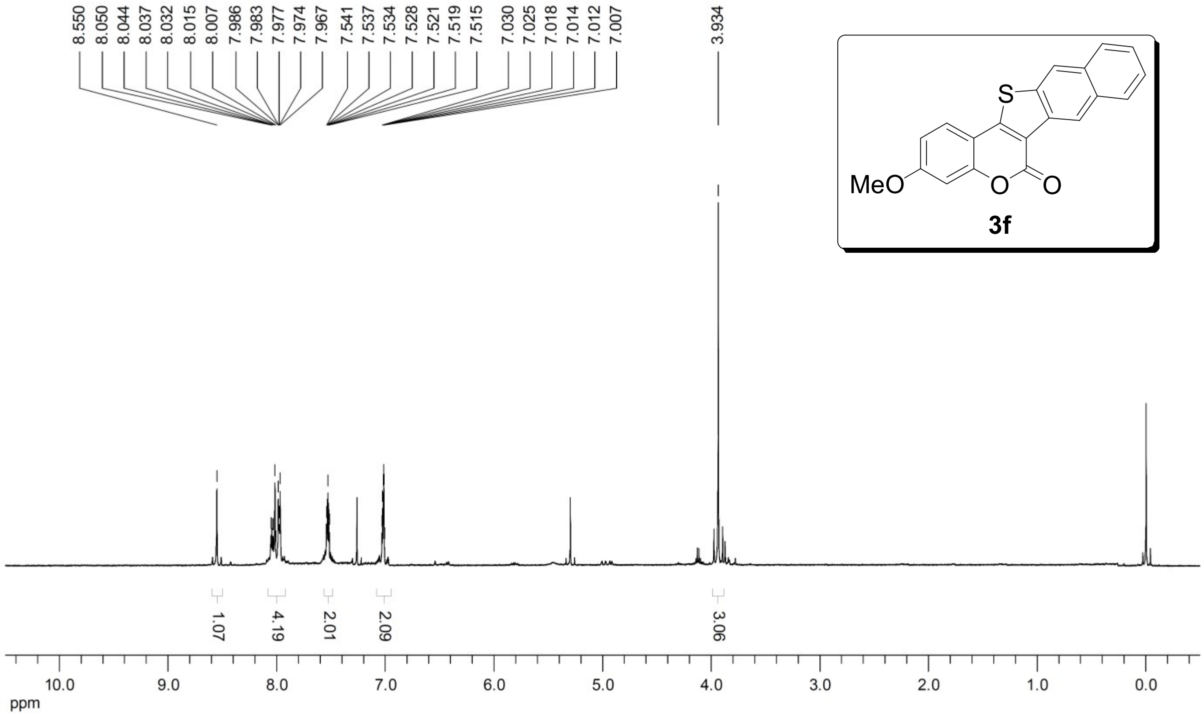

Figure 11. **3f** ^{1}H NMR

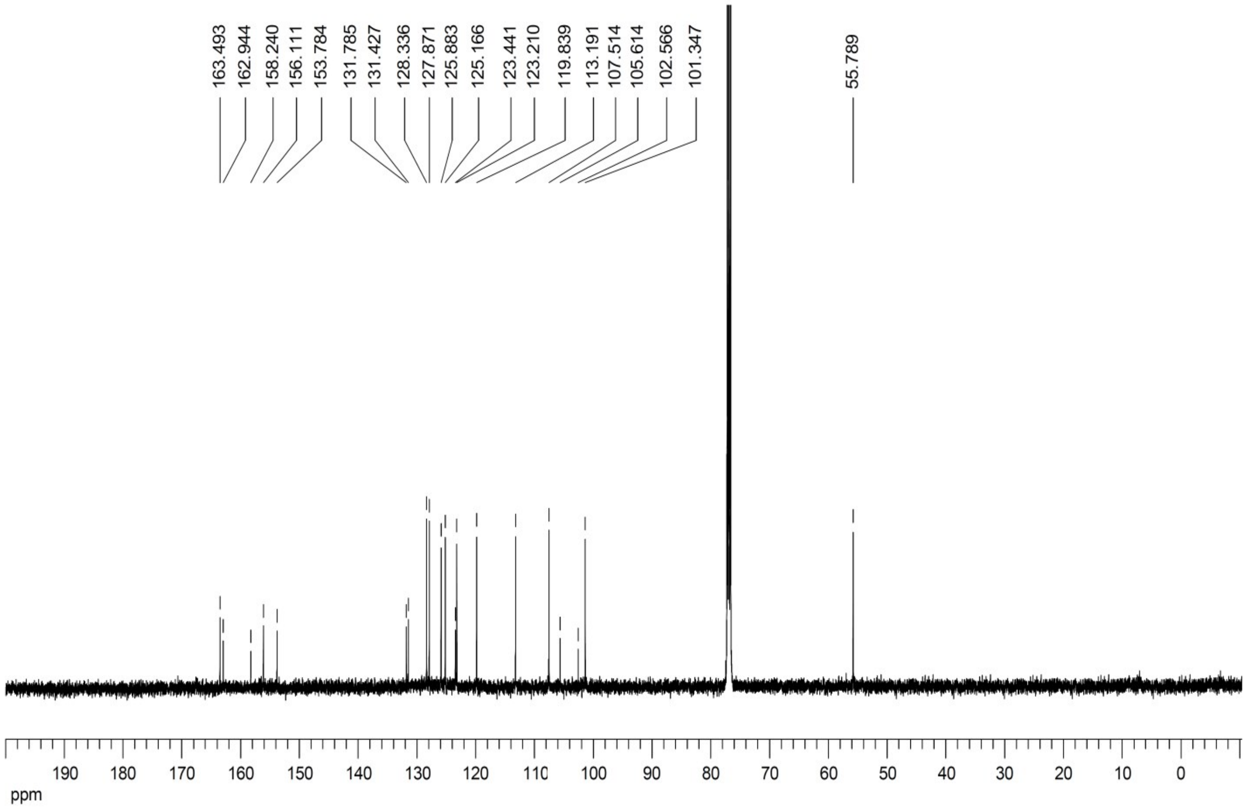

Figure 12. **3f** ^{13}C NMR

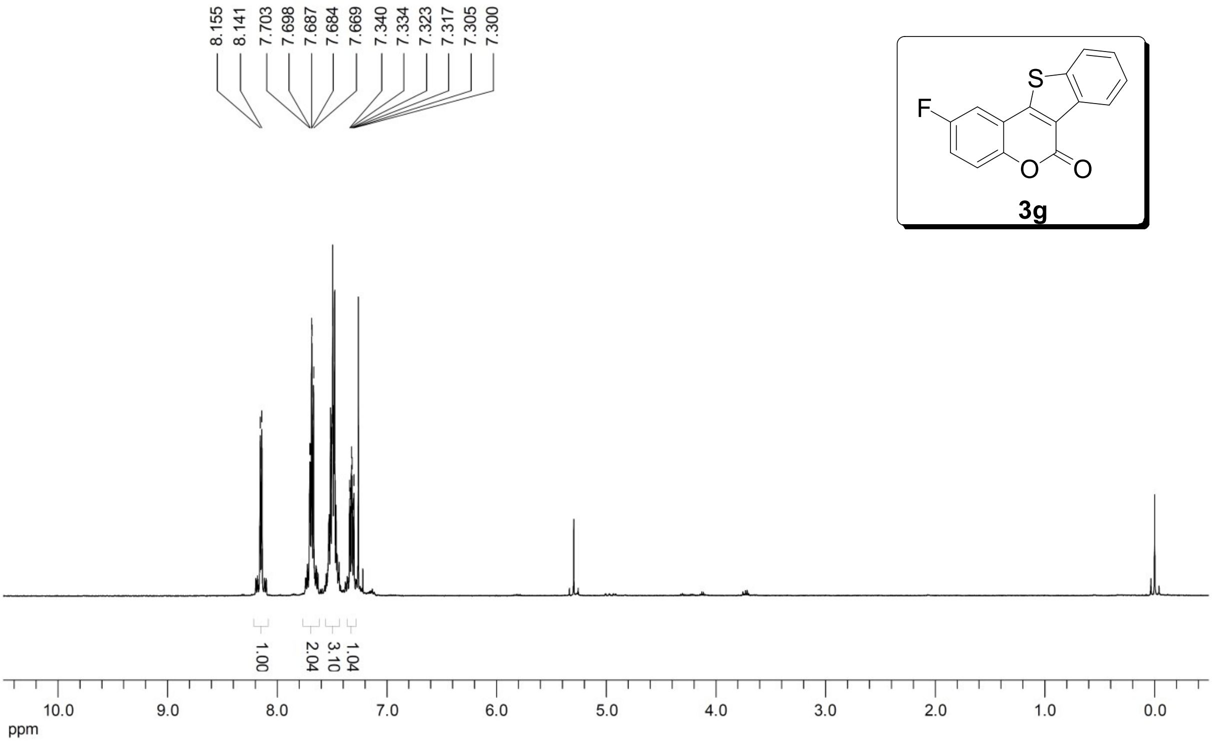

Figure 13. **3g** ^{1}H NMR

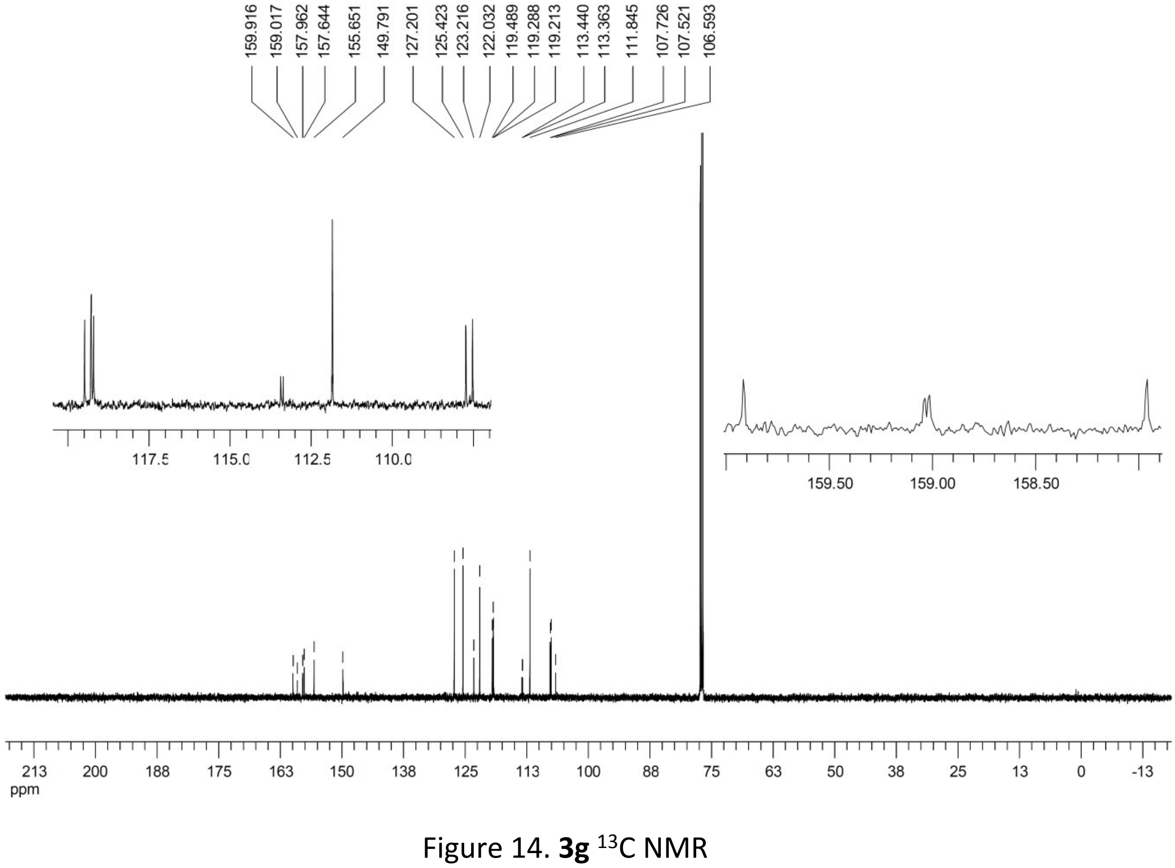

Figure 14. **3g** ^{13}C NMR

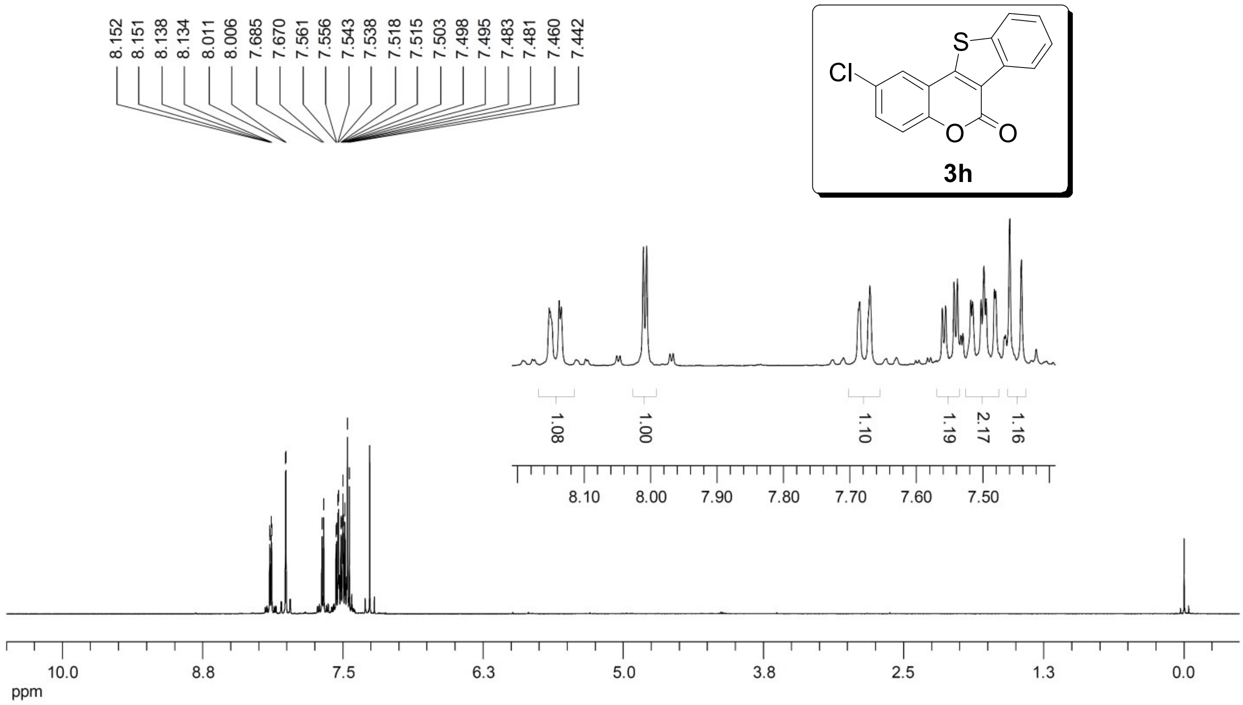

Figure 15. **3h** ^{1}H NMR

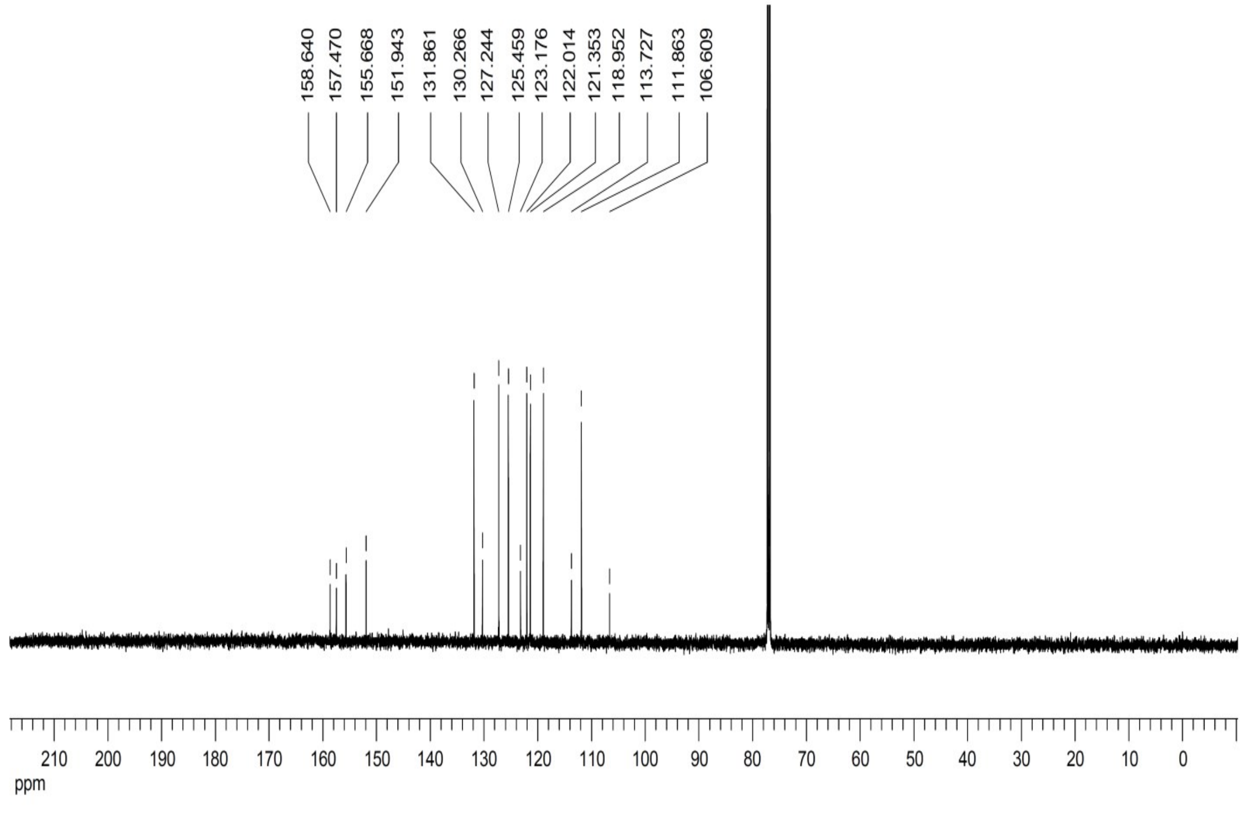

Figure 16. **3h** ^{13}C NMR

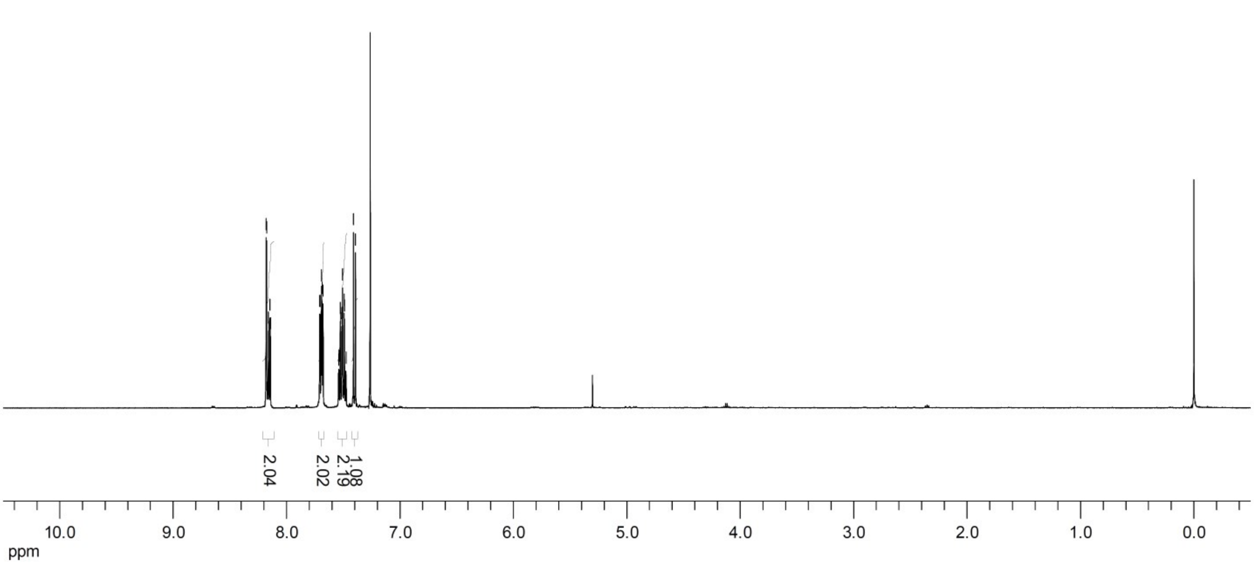

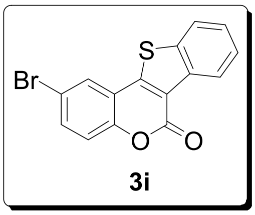

Figure 17. **3i** ^1H NMR

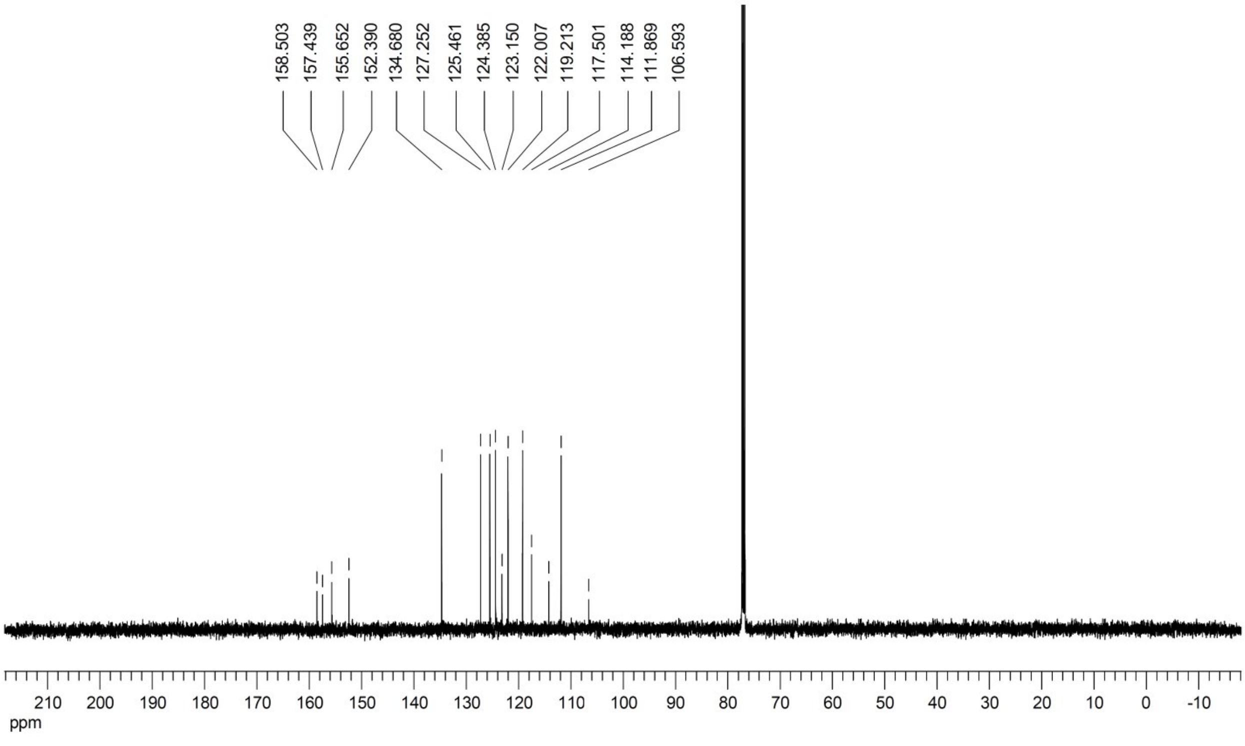

Figure 18. **3i** ^{13}C NMR

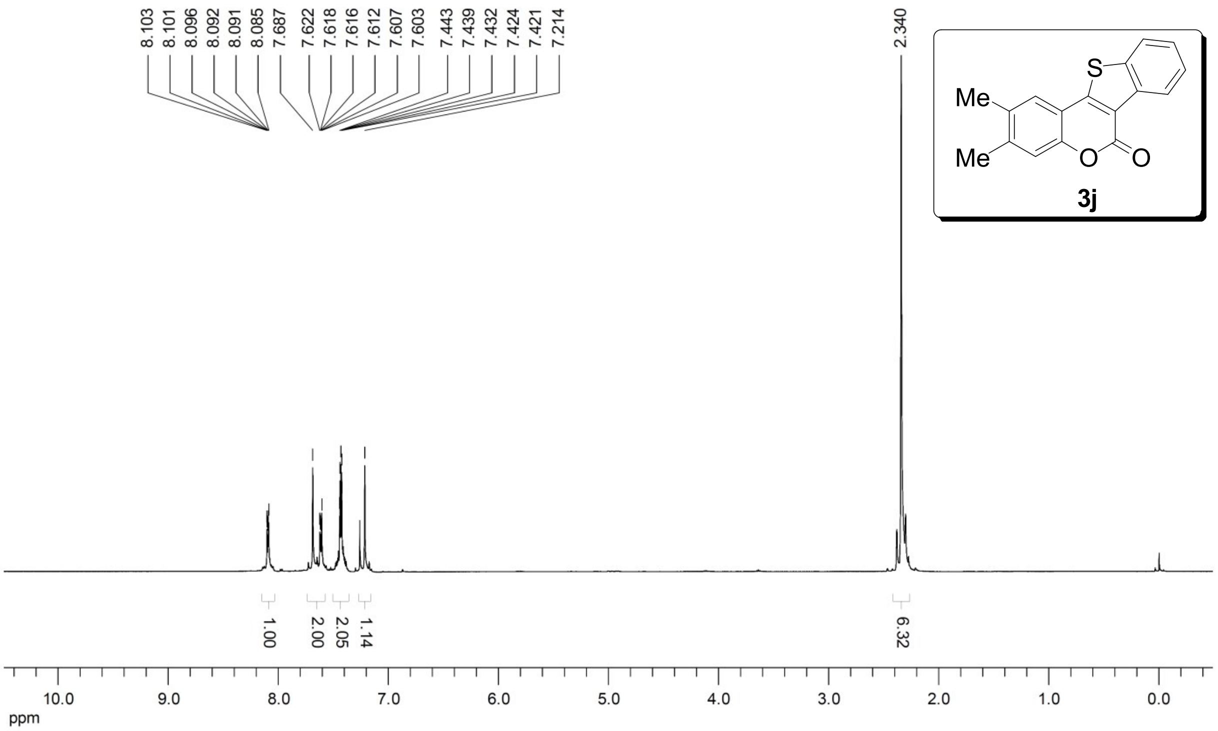

Figure 19. **3j** ^{1}H NMR

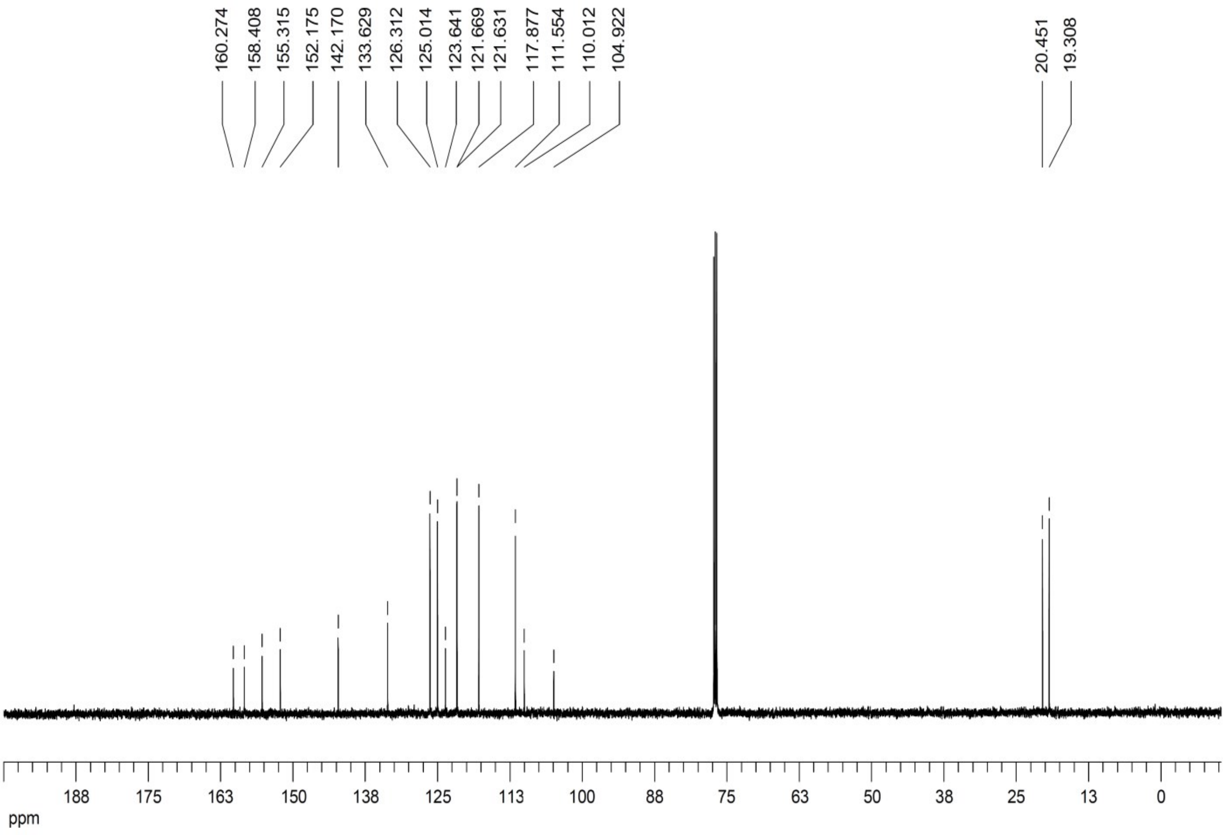

Figure 20. **3j** ^{13}C NMR

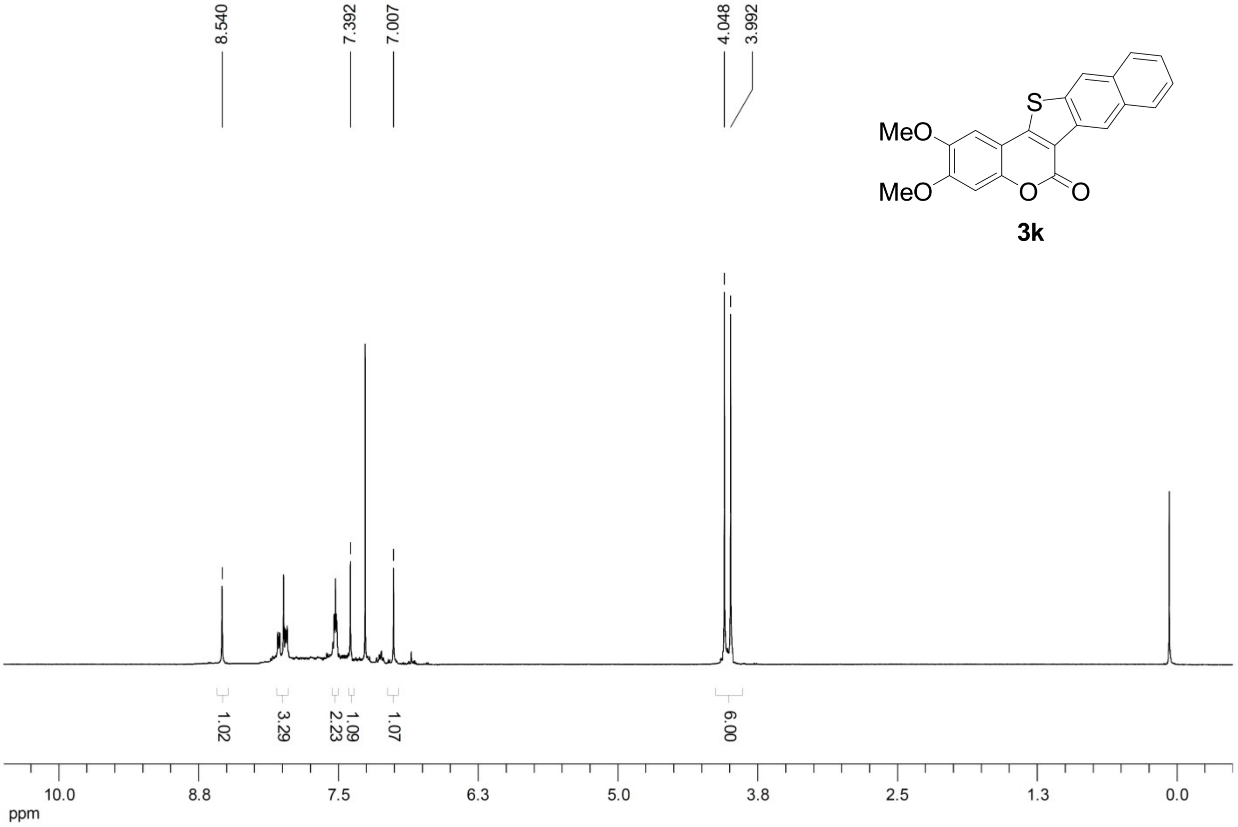

Figure 21. **3k** ^{1}H NMR

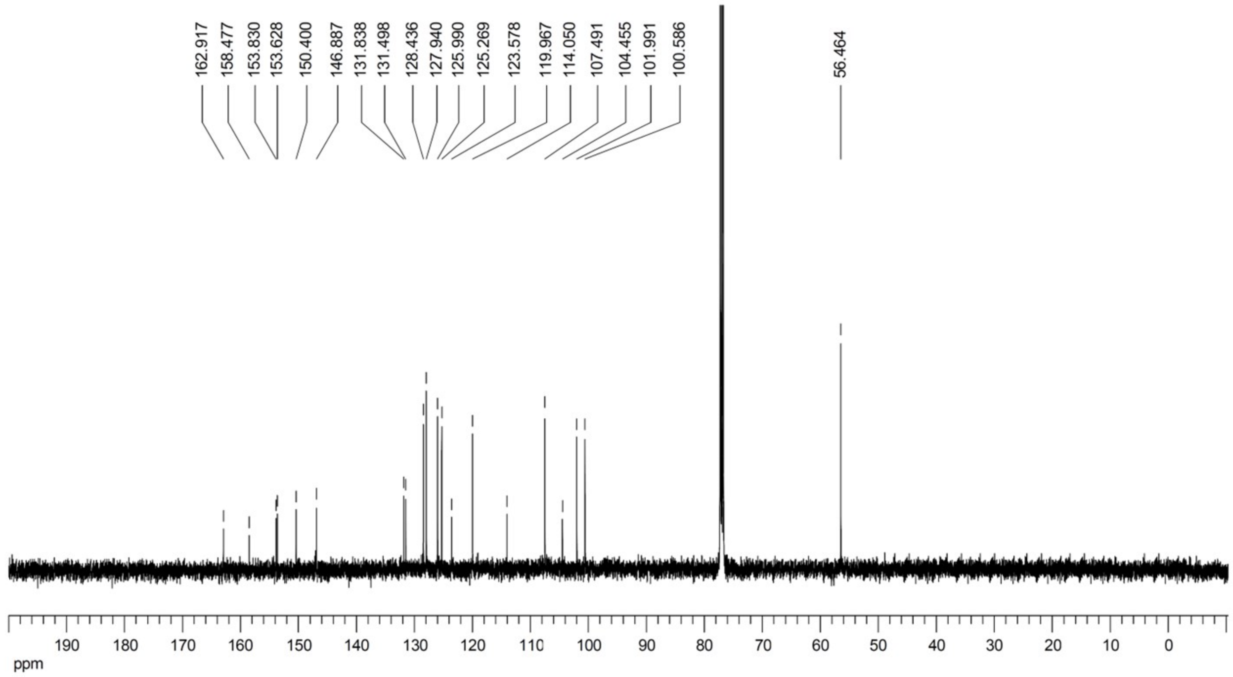

Figure 22. **3k** ^{13}C NMR

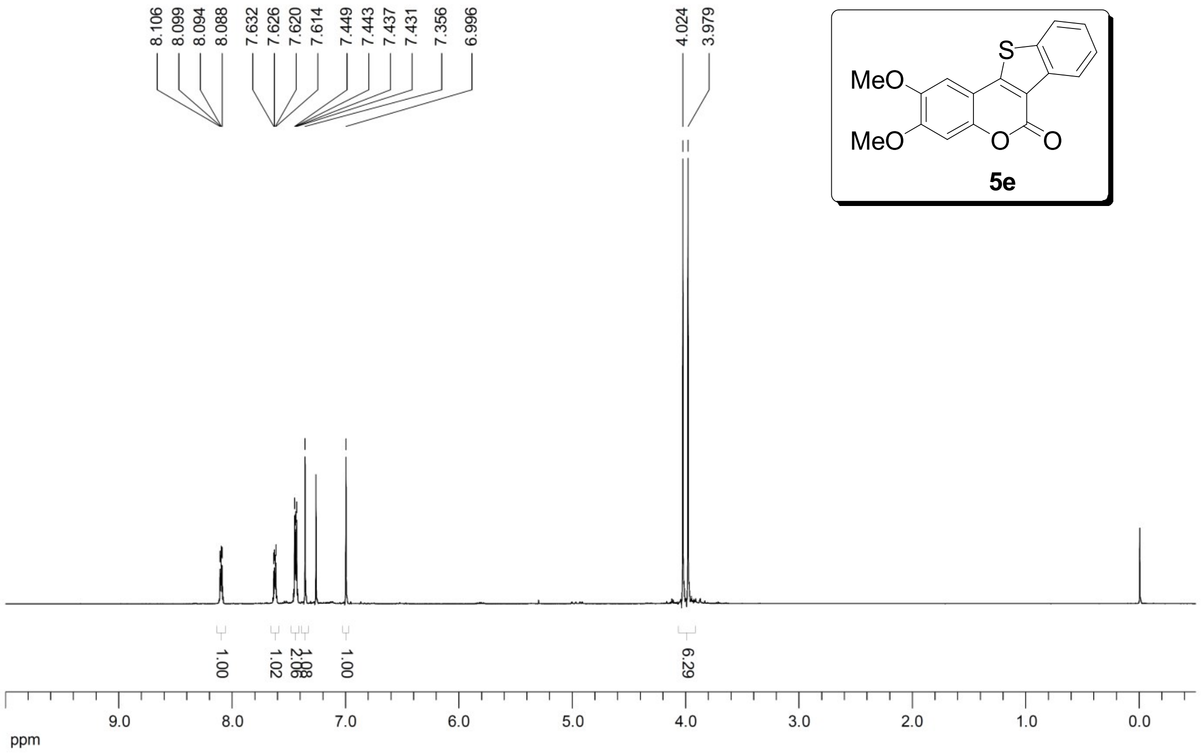

Figure 23. **5e** ^{1}H NMR

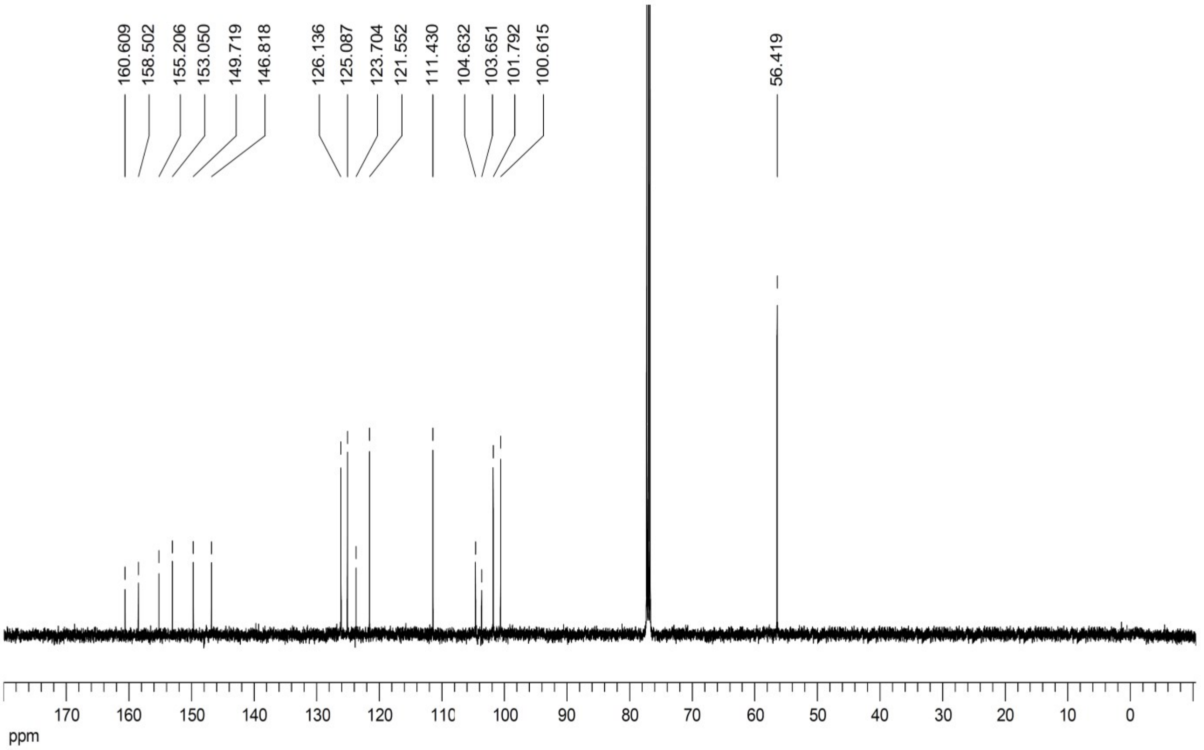

Figure 24. **5e** ^{13}C NMR
